# MoS_2_ phononic crystals for advanced thermal management

**DOI:** 10.1126/sciadv.adm8825

**Published:** 2024-03-29

**Authors:** Peng Xiao, Alexandros El Sachat, Emigdio Chávez Angel, Ryan C. Ng, Giorgos Nikoulis, Joseph Kioseoglou, Konstantinos Termentzidis, Clivia M. Sotomayor Torres, Marianna Sledzinska

**Affiliations:** ^1^ Catalan Institute of Nanoscience and Nanotechnology (ICN2), CSIC and BIST, Campus UAB, Bellaterra, 08193 Barcelona, Spain.; ^2^ Departamento de Física, Universidad Autónoma de Barcelona, Bellaterra, 08193 Barcelona, Spain.; ^3^ National Center for Scientific Research “Demokritos,” 15310 Athens, Greece.; ^4^ Department of Physics, Aristotle University of Thessaloniki, GR-54124 Thessaloniki, Greece.; ^5^ Center for Interdisciplinary Research and Innovation, Aristotle University of Thessaloniki, Thessaloniki, Greece.; ^6^ Univ Lyon, CNRS, INSA Lyon, CETHIL, UMR5008, 69621 Villeurbanne, France.; ^7^ ICREA, Passeig Lluis Companys 23, 08010 Barcelona, Spain.

## Abstract

Effective thermal management of electronic devices encounters substantial challenges owing to the notable power densities involved. Here, we propose layered MoS_2_ phononic crystals (PnCs) that can effectively reduce thermal conductivity (κ) with relatively small disruption of electrical conductivity (σ), offering a potential thermal management solution for nanoelectronics. These layered PnCs exhibit remarkable efficiency in reducing κ, surpassing that of Si and SiC PnCs with similar periodicity by ~100-fold. Specifically, in suspended MoS_2_ PnCs, we measure an exceptionally low κ down to 0.1 watts per meter kelvin, below the amorphous limit while preserving the crystalline structure. These findings are supported by molecular dynamics simulations that account for the film thickness, porosity, and temperature. We demonstrate the approach efficiency by fabricating suspended heat-routing structures that effectively confine and guide heat flow in prespecified directions. This study underpins the immense potential of layered materials as directional heat spreaders, thermal insulators, and active components for thermoelectric devices.

## Teaser

2D phononic crystals enable efficient heat management in nanoelectronics.

## INTRODUCTION

The development of three-dimensional (3D) integrated circuits (ICs) has brought notable challenges in power and thermal management due to their higher power density compared to traditional 2D chips (*1*). Effectively managing the excess heat generated by these devices is of paramount importance. Consequently, recent research efforts have been devoted to control and manipulate heat transport at the nanoscale (*2*–*7*). In particular, effective temperature regulation of hotspots in high-speed circuits is essential as these localized regions affect device performance and reliability, ultimately determining thermal design considerations. A potential avenue to advance current thermal engineering solutions lies in the integration of 2D materials within 3D ICs. These materials exhibit strong anisotropic thermal conductivity. For instance, layered crystalline MoS_2_, one of the most extensively investigated transition metal dichalcogenides (TMDs), exhibits a factor of 10 anisotropy ratio between in-plane and cross-plane thermal conductivity (*8*–*14*). This anisotropic behavior indicates that MoS_2_ can expeditiously dissipate heat in the in-plane direction without adversely affecting other vertically aligned chips in its proximity. By contrast, conventional 3D isotropic heat spreaders such as copper plates cannot achieve a similar type of directional heat dissipation. Moreover, other 2D materials, such as hBN and graphene, which have an intrinsically high κ (*15*), exhibit similar heat guiding functionalities but cannot be used as active materials in electronic devices.

In this context, with the ability to direct heat along a precisely defined path through a combination of blocking and subsequent guiding of heat, one can envisage air-stable, 2D layered materials for heat-routing structures in 3D ICs (*16*, *17*). This would enable the dissipation of heat away from hotspots, mitigating any potential damage to subjacent structures. Since 2D materials have the inherent advantage of limited heat transfer in the cross-plane direction due to the weak van der Waals interactions between individual layers, modification of the κ could allow effective in-plane heat flow control. Moreover, TMDs have attracted notable attention as promising thermoelectric materials (*18*, *19*) capable of harvesting electrical energy from temperature gradients. The additional patterning of 2D semiconductors could enable in-plane heat guiding and increased heat dissipation.

In general, the reduction of κ in 2D materials can be achieved by introducing defects or grain boundaries (*20*, *21*). Other approaches, such as nanopatterning by electron beam lithography or focused ion beam (FIB) can also be used to produce 2D PnC structures, where the periodic patterning of holes results in a reduction of κ due to increased phonon boundary scattering (*22*–*25*). Nanostructuring techniques also have shown promising results in 3D materials such as silicon by reducing the acoustic phonon mean free path (MFP) (*26*, *27*). Nevertheless, there is a lack of similar studies on thermal transport engineering, particularly in 2D semiconductor materials. In the case of MoS_2_, theoretical investigations suggest that the phonon MFP spans a range from 5 to 20 nm (*8*, *11*, *28*). However, a recent theoretical study indicates that nanostructures with a periodicity of about 400 nm can yield a substantial reduction in the κ of monolayer MoS_2_ (*29*).

In this work, we first demonstrate the efficient reduction of κ in freestanding MoS_2_ membranes using FIB nanopatterning. The functionality and underlying mechanisms of heat transfer in these structures are supported by thermal transport experiments and equilibrium molecular dynamics (EMD) simulations in both few-layer pristine and nanopatterned MoS_2_ consisting of periodically arranged holes of varying periods (100 to 500 nm). Specifically, the κ of all the MoS_2_ membranes in this study were determined by two-laser Raman thermometry (2LRT) and one-laser Raman thermometry (1LRT). The effect of sample thickness and temperature on the κ of MoS_2_ and MoS_2_ PnCs were calculated by EMD simulations based on the autocorrelation function of the heat flux. The findings reveal that periodic arrays of holes, even with periods larger than the average phonon MFP, substantially reduce the κ with relatively small disruption of the σ. Furthermore, this study reports the first realization of heat routing structures nanopatterned in layered 2D materials including a thermal insulating ring, which confines heat in a delimited area, and a heat guiding channel, which confines heat and conducts it away from a hotspot in the in-plane direction. This straightforward approach, which can direct the heat flow through arbitrary paths, is a promising thermal management strategy for various applications such as 3D ICs and potentially for thermoelectric generators and other nanoelectronics.

## RESULTS AND DISCUSSION

**Fig. 1.**
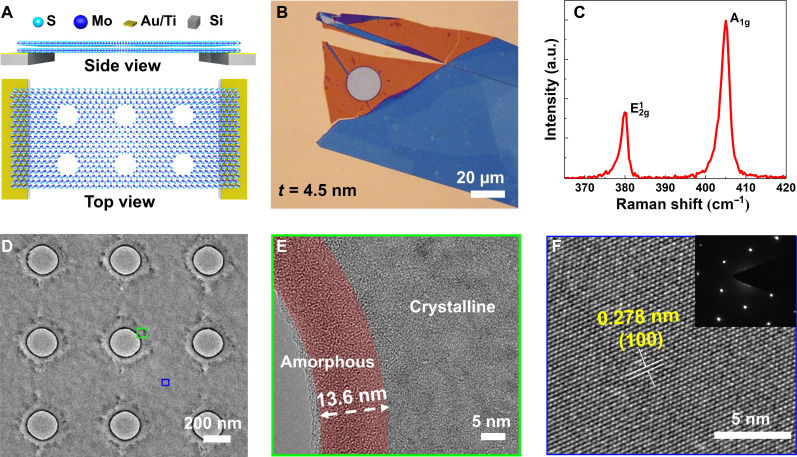
Characterization of freestanding MoS_2_ phononic crystals. (**A**) Side and top schematic of the MoS_2_ crystal structure, in which Mo atoms (blue spheres) are sandwiched between sulfur atoms (light blue spheres). The left and right sides of the membrane were placed on a Au (95 nm)/Ti (5 nm)/Si TEM substrate with holes. (**B**) Optical image of a 4.5-nm-thick MoS_2_ membrane. (**C**) Raman scattering spectrum of the MoS_2_ membrane obtained with a 532-nm laser. (**D**) TEM image of the 4.5-nm-thick MoS_2_ PnC membrane was nanopatterned by FIB with a hole period of 500 nm. (**E**) A high-resolution TEM image of the hole edge of the region corresponding to the green square in (D). (**F**) A high-resolution TEM image of the crystalline neck of the region corresponding to the blue square in (D) and the corresponding electron diffraction pattern. a.u., arbitrary units.

The MoS_2_ samples were prepared using mechanical exfoliation and subsequent dry transfer techniques. Schematics of the side and top views of the crystalline MoS_2_ with PnCs structure are shown in Fig. 1A. The morphological and structural characterization of the samples was performed using optical microscopy, Raman scattering spectroscopy, high-resolution scanning transmission electron microscopy (TEM), and atomic force microscopy (AFM). A representative optical image of the 4.5-nm-thick MoS_2_ membrane is shown in Fig. 1B. The crystalline nature of MoS_2_ was confirmed through Raman spectroscopy as shown in Fig. 1C, in agreement with previous reports (*30*). The thickness of the samples was obtained from tapping mode AFM measurements (details in fig. S1).

**Fig. 2.**
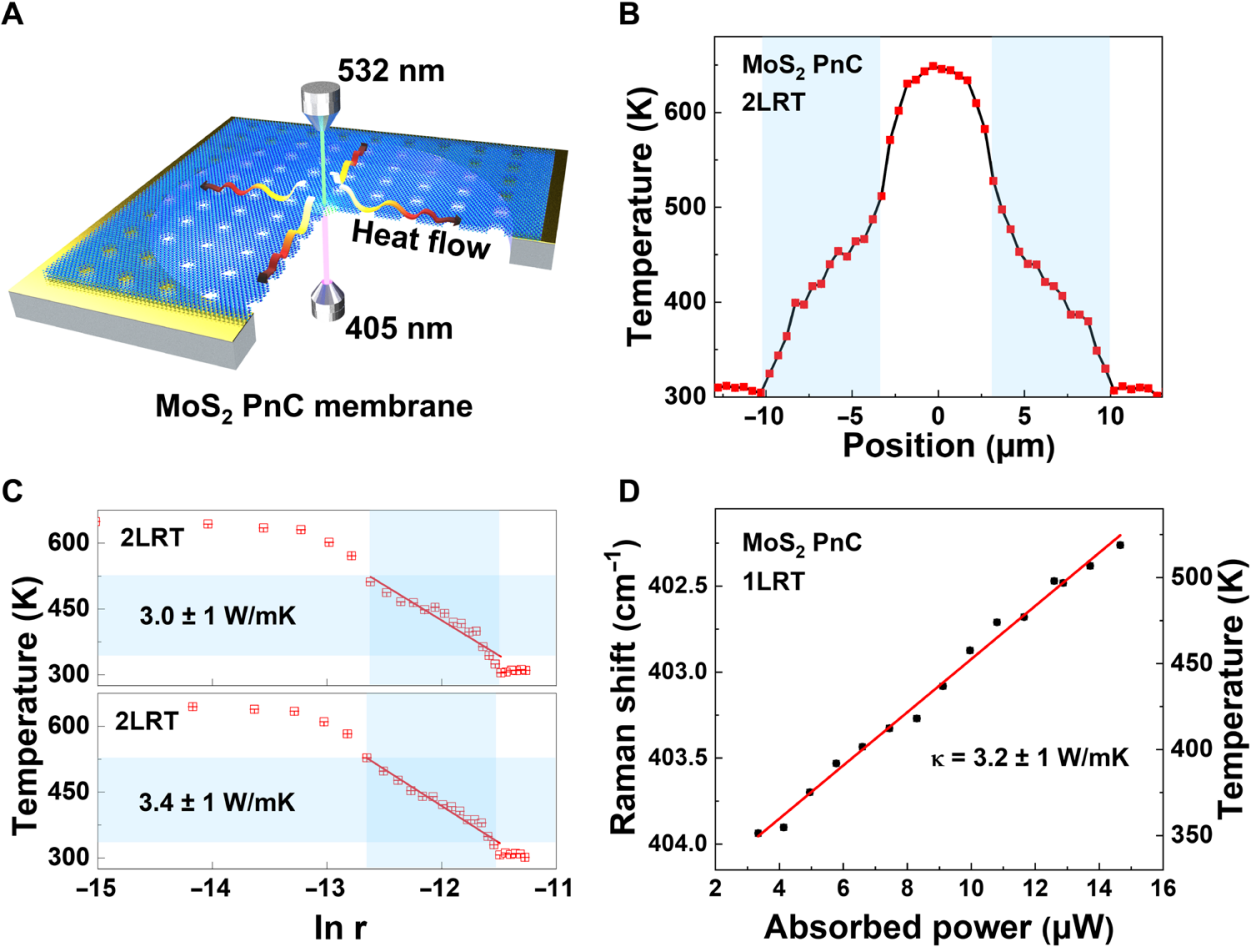
Thermal characterization of patterned freestanding MoS_2_ membranes. (**A**) Schematic of a thermal conductivity measurement of a MoS_2_ PnC membrane by the 2LRT setup, in which a heating laser (405 nm) was focused onto the membrane center and a probe laser (532 nm) used to scan straight through the center point. (**B**) Temperature profile over the sample extracted using the 2LRT line scan measurement and (**C**) the corresponding left and right sides from the center temperature profiles as a function of ln (*r*). The solid lines show the linear fits of the experimental points. (**D**) The Raman shift versus absorbed power measured in 1LRT experiments. The results were matched to the COMSOL simulated data (red line).

After conducting κ measurements on the pristine MoS_2_ membranes, PnCs were fabricated within these membranes with periods ranging from 100 to 500 nm. Using the Ga + ion FIB, a prevalent tool with high process efficiency in nanofabrication facilities, nanopatterning was performed in this study. The dimensions of the FIB-fabricated PnCs were characterized using TEM and scanning electron microscopy (see Fig. 1D and fig. S2). The 30-kV Ga^+^ ion beam can be approximated as a Gaussian shape. The ion exposure can cause partial damage to the non-milled material, resulting in increased surface roughness and an introduction of defects in the crystalline structure that could affect the properties of the MoS_2_ (*24*). To minimize the damage, a low gallium ion current of 2 pA was used. Even so, an amorphous region with a width of approximately 10 to 30 nm resulting from local FIB damage surrounding each hole in the TEM image was observed (Fig. 1E). The remaining area between holes maintained its crystalline structure, as confirmed by TEM and electron diffraction examination (Fig. 1F and fig. S3). The amorphous dimensions of other samples were obtained from TEM images (fig. S4). As expected, for the same exposure time, the hole diameter decreased with increasing MoS_2_ thickness, while the width of the amorphous region slightly increased with increasing thickness (fig. S5). Furthermore, the gallium atom trace acquired through TEM energy dispersive spectroscopy revealed the restricted influence of gallium doping on MoS_2_, as illustrated in fig. S6. The 2LRT and 1LRT setups (*20*, *31*–*33*) were used to determine the κ of these samples, and both were done to achieve an accurate measurement by two separate and independent methods. The 2LRT configuration is illustrated in Fig. 2A. A heating laser (405 nm) shone onto to the sample from below generates a temperature gradient that induces shifts in the frequencies of the Raman active modes of MoS_2,_ namely, E^2^_g_ and A_1g_. The Raman spectra were measured by a low-power probe laser (532 nm) focused on the top of the samples, which coupled the scattered light to the Raman spectrometer. All shifts in Raman peak positions were then converted to temperature changes using pre-established calibration curves (fig. S7 and table S1). Because of the system symmetry, a Raman spectra line scan can be collected in 2LRT when the probe laser linearly scans the freestanding membrane across its diameter (Fig. 2, B to D) (*20*, *27*, *34*). In 1LRT configuration, with increasing laser intensity of the 532-nm laser, which was focused on the center of the MoS_2_ membrane, the relation between temperature and absorbed laser power was measured and matched to the COMSOL simulation to extract the value of κ. In contrast, in 2LRT experiments, the temperature distribution on the freestanding membranes was determined using 2D thermal maps. However, these cannot be used for the MoS_2_ PnCs with periods <200 nm due to the effect of holes on the Raman signals. Specifically, with the reduction in the period of PnCs, there is a noticeable shift in the Raman peak position and a substantial decrease in the intensity of the Raman spectra (fig. S7). The spectra show a very weak signal-to-noise ratio when the MoS_2_ PnCs period falls below around 200 nm. Therefore, using 1LRT experiments for all the samples, the κ was obtained from the COMSOL model. Details on the experimental methodology and κ calculations are described in Materials and Methods*.*

Thermal transport in freestanding layered membranes is dominated by the in-plane κ, which can be calculated using Fourier’s law: *P*/(2π*rt*) = −κ*dT*/*dr*, where *P* is the power absorbed by the membrane, *r* is the distance from center, and *t* is the membrane thickness (*27*, *31*). By taking *rdT*/*dr* = *dT*/*d*(*lnr*) the following expression for κ was obtained: $\kappa =-P/\left[2\pi t\frac{dT}{d\left(lnr\right)}\right]$ . Figure 2B shows the heating scan curve measured by 2LRT, which demonstrates a symmetric temperature decay from the center of the sample toward the heat sink (membrane edge), where it reaches room temperature. The profiles are well fitted with a constant κ, as depicted in Fig. 2C, where the slope of the fitting lines corresponds to *dT*/*d*(*lnr*). We note that the background scan curves exhibit changes in the A_1g_ peak frequency before and after nanopatterning (fig. S8), which can be attributed to size effects (*35*). Figure 2D shows the 1LRT measurement results, which were compared to the simulated data from the COMSOL model (fig. S8F) to obtain a final value of κ. To extract the intrinsic κ of the MoS_2_ PnCs, the experimental value was corrected using a volume correction factor ε, which takes into account the volume reduction of the patterned samples, where ε = (1 − δ)/(1 + δ), and where the porosity δ is given by the equation δ = π*d*^2^/4*a*^2^ (*27*, *36*).

**Fig. 3.**
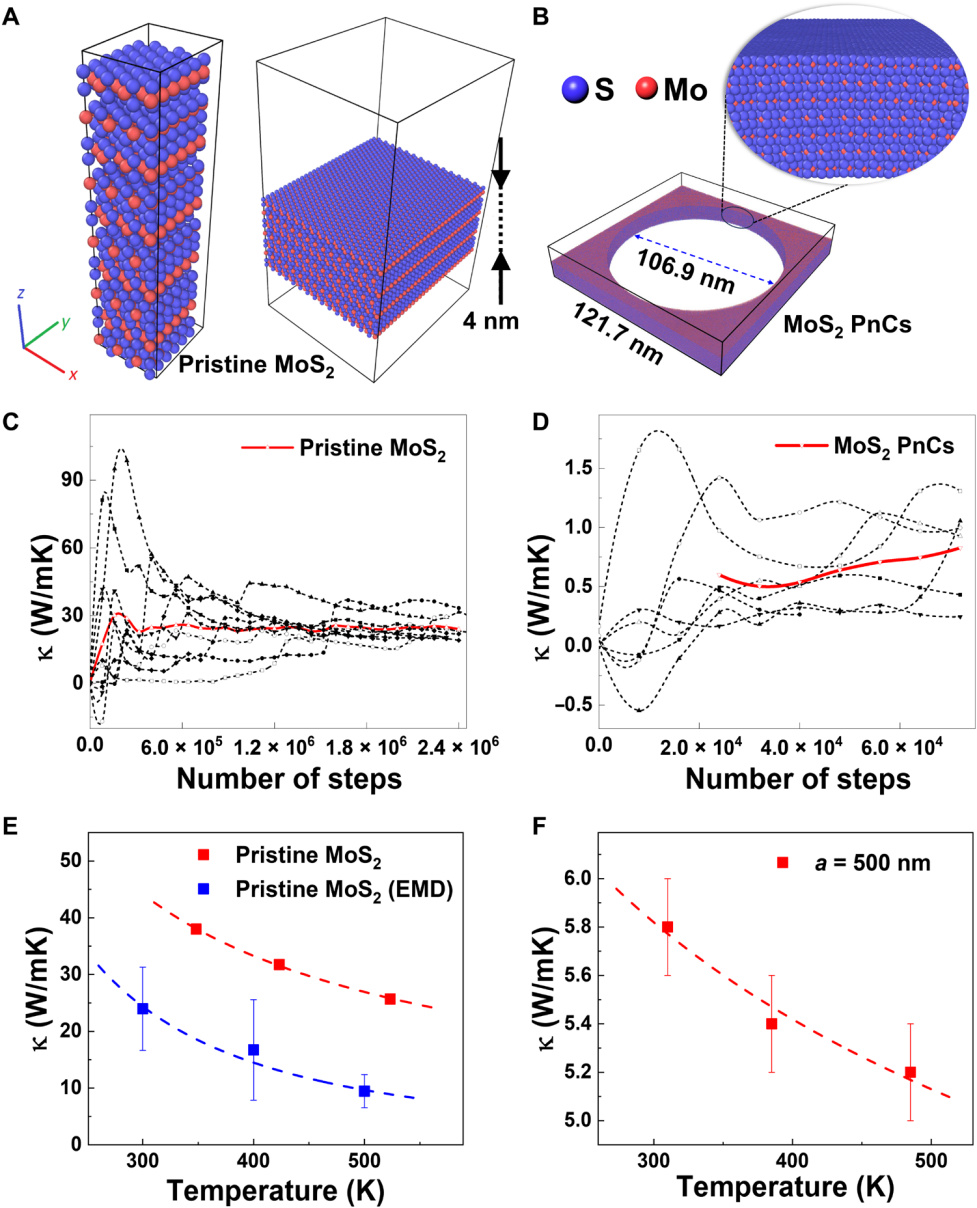
MD simulations and temperature-dependent thermal conductivity of MoS_2_. (**A**) and (**B**) show the atomistic configurations of the pristine MoS_2_ membrane and the 11-nm-thick MoS_2_ PnCs membrane, respectively. (**C**) and (**D**) show the EMD simulated κ_in-plane_ results corresponding to the pristine MoS_2_ and MoS_2_ PnCs, and the mean values were calculated using 10 random seeds. (**E**) Comparison between 1LRT-based experiment (12-nm-thick MoS_2_) and EMD simulations (10-nm-thick MoS_2_) of temperature-dependent κ of a freestanding MoS_2_ membrane. (**F**) The 1LRT-based experimental (12-nm-thick MoS_2_) temperature-dependent κ of the same membrane after FIB nanopatterning. Dashed lines are guides to the eye.

To gain further insights into the impact of thickness and patterning on κ, we performed EMD simulations (*37*). κ was studied with the EMD method using the Large-scale Atomic/Molecular Massively Parallel Simulator package and the Reactive Empirical Bond Order–Lennard Jones potential (*38*), which indicated that the κ of MoS_2_ increased from approximately 9 to 24 W/mK as the thickness was increased from 4 to 10 nm. The κ of 10-nm-thick MoS_2_ reached 62% of the bulk value. These simulations were compared to the experimental data from the 11-nm-thick MoS_2_ PnCs, which have the lowest intrinsic κ achieved in this study. The EMD model of the pristine MoS_2_ and MoS_2_ PnCs is illustrated in Fig. 3 (A and B). The sulfur atoms are irregularly arranged on the hole surface in the MoS_2_ PnCs due to their greater mobility relative to the molybdenum atoms (*39*). The whole surface was partially covered with sulfur atoms. The EMD simulations indicated that the κ of the 11-nm-thick MoS_2_ membranes with a period of 122 nm was 0.85 ± 0.50 W/mK, which is on the same magnitude as the experimental value (0.2 ± 0.1 W/mK). The qualitative agreement between the experimental and theoretical results validates our approach, a strategy that can be readily extended to other patterned layered materials.

Moreover, from the nonequilibrium MD simulations, we calculated the phonon MFPs in the in-plane direction of the MoS_2_ sample to be approximately 41 nm in a 4-nm-thick MoS_2_ (fig. S14) (*40*). Using the MFP reconstruction method, an MFP of 30 nm was obtained for bulk MoS_2_. This method was applied to the experimental results assuming that the diffusive thermal transport is governed by the Fuchs-Sondheimer approach (fig. S14) (*31*, *41*–*45*). In addition, we studied the temperature dependence of the κ of a 10-nm-thick MoS_2_ membrane_._ Average temperature values were used for κ determination. Through this method, both experimental and theoretical results closely align the expected T^−1^ trend. Both experiment and simulations showed that the κ decreases with increasing temperature, as shown in Fig. 3D. Figure 3E shows that MoS_2_ PnCs also exhibit the same temperature-dependent properties. For pristine MoS_2_ membranes, this effect originates from the temperature dependence of the phonon MFP; e.g., with increasing temperature, the wavelength of phonons dominating the heat transport becomes shorter, and phonon-phonon scattering (Umklapp processes) becomes dominant (*31*, *46*). In contrast, for the MoS_2_ PnCs, the inclusion of an extra scattering mechanism (hole scattering) together with the surface overwhelms the phonon-phonon processes leading to the smaller temperature dependence of thermal conductivity (*47*).

**Fig. 4.**
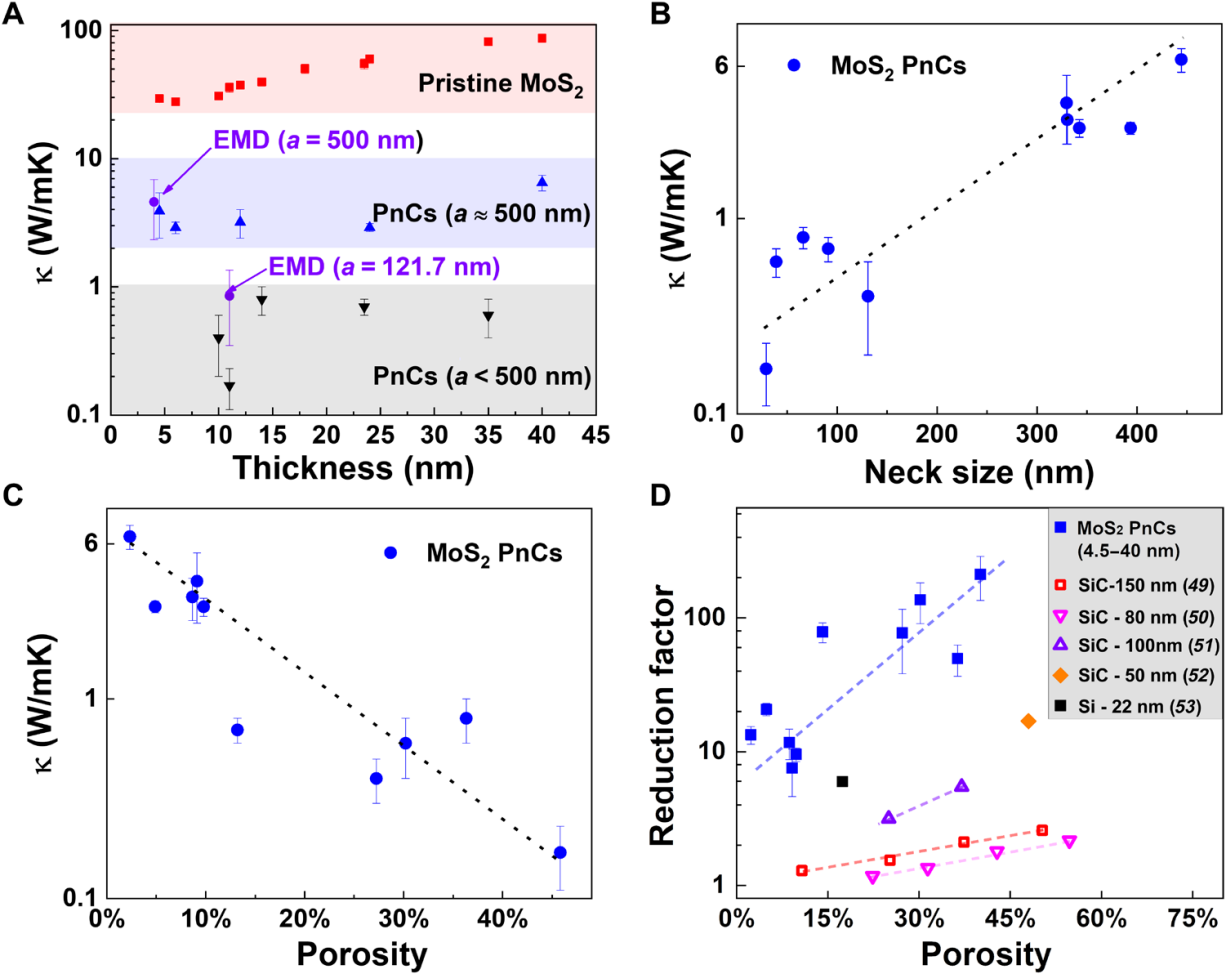
Reduction of the thermal conductivity of crystalline MoS_2_ membranes. (**A**) Comparison of the experimental κ_in-plane_ of the pristine and nanopatterned MoS_2_ PnCs, with the EMD simulated κ. (**B**) and (**C**) show the κ of MoS_2_ PnCs with different periods as a function of neck size and porosity, respectively. (**D**) Comparison of the reduction factor as a function of porosity for MoS_2_ in this work and other nanopatterned semiconducting membranes (*49*–*53*).

Two sets of samples were designed and measured to understand the effect of membrane thickness and PnC periodicity on the thermal reduction efficiency. The reduction efficiency is defined by a reduction factor of *R*_κ_ = κ_pristine_/κ_p-int_, where κ_pristine_ is the thermal conductivity of a pristine 2D membrane and κ_p-int_ is the intrinsic thermal conductivity of a 2D MoS_2_ PnC. These results are compared to the original κ and the MD simulated data, as shown in Table 1. The first set of samples contains PnCs with a period of about 500 nm to study the effect of thickness on the thermal reduction efficiency in the 2D layered system. The second set of PnC samples had smaller periods and was used to study the effects of neck size and porosity on κ of the MoS_2_ PnCs. The information is graphically represented in Fig. 4 to facilitate comparison and further analysis. The dependence of κ_in-plane_ on the thickness in MoS_2_ is shown in Fig. 4A. In pristine MoS_2_, κ_pristine_ increases almost linearly from 29.4 ± 1.5 to 86.9 ± 4.8 W/mK for thicknesses from 4.5 to 40 nm. The minimum value of κ_pristine_ measured in the 6-nm-thick sample can be explained considering two competing scattering mechanisms, i.e., phonon-phonon and surface-phonon scattering, as has been previously reported in supported six to eight layers of MoS_2_ (*8*). By comparing the κ_pristine_ and κ_p-int_ of PnCs with period *a* = 500 nm, we found that the reduction factor *R*_κ_ for PnCs is approximately 10 as the thickness increases from 4.5 to 40 nm. Notably, κ_p-int_ and κ_pristine_ exhibited a consistent dependence on thickness, with all showing a small dip in κ_pristine_ for the 6-nm-thick sample. The thermal conductivity of amorphous materials is typically around 0.1 W/mK, while the thermal conductivity of our MoS_2_ PnCs is larger than this value (*10*). This observation serves as an additional validation, reaffirming the preservation of the layered structure of MoS_2_ following the FIB nanopatterning process. In addition, the σ_pristine_ of a layered MoS_2_ was characterized using the four-probe method under the flow of nitrogen (fig. S10). After patterning PnC with the typical period of 500 nm using Ga^+^ FIB, the sample’s σ_pnc_ was measured with the same conditions. As shown in table S2, the σ reduction factor *R*_σ_ for a 118-nm-thick MoS_2_ PnC (*a* = 500 nm) is ~2.1.

**Table 1.** Parameters for the MoS_2_ PnC samples. *t* is the membrane thickness, *a* is the PnC period measured as the center-to-center distance between the holes, *d* is the hole diameter, *n*_a_ is the amorphous area size, κ_p-eff_ is the effective κ, κ_p-int_ is the intrinsic κ, and *R*_κ_ represents the thermal conductivity reduction factor, which is defined by *R*_κ_ = κ_pristine_/κ_p-int_.

*t* (nm)	*a* (nm)	*d* (nm)	*n_a_* (nm)	κ_p-eff_ (2LRT) (W/mK)	κ_p-eff_ (1LRT) (W/mK)	κ_p-int_ (W/mK)	κ_pristine_ (2LRT) (W/mK)	*R*_κ_
4 (EMD)	500	315	/	/	/	4.6 ± 2.0	/	/
4.5	500	170	13.6	3.2 ± 1.2	3.2 ± 0.2	3.9 ± 1.5	29.4 ± 1.5	7.5
6	529	187	11.6	2.3 ± 0.2	2.1 ± 0.1	2.9 ± 0.3	27.7 ± 1.5	9.6
10	318	187	11.5	0.2 ± 0.1	/	0.4 ± 0.2	30.9 ± 2.0	77.3
11	122	93	9.9	/	0.06 ± 0.02	0.17 ± 0.06	36.0 ± 3.0	211.8
11 (EMD)	121.7	106.9	/	/	/	0.85 ± 0.50	/	/
12	494	164	21.7	2.7 ± 0.7	4.0 ± 0.4	3.2 ± 0.8	37.5 ± 1.9	11.7
14	205	139	20	/	0.4 ± 0.1	0.8 ± 0.2	39.7 ± 3.0	49.6
18	/	/	/	/	/	/	50.3 ± 4.0	/
23.5	154	63	23.3	/	0.5 ± 0.1	0.7 ± 0.1	55.0 ± 5.0	78.6
24	524	131	22.7	2.6 ± 0.2	5.4 ± 0.5	2.9 ± 0.2	60.1 ± 4.8	20.7
35	106	67	/	/	0.3 ± 0.1	0.6 ± 0.2	82.0 ± 3.0	136.7
40	537	93	24.5	6.2 ± 0.9	9.4 ± 0.5	6.5 ± 0.9	86.9 ± 4.8	13.4

Furthermore, MoS_2_ PnCs with smaller periods (100 to 300 nm) were fabricated to investigate the role of neck size and porosity on κ in such engineered 2D materials. It was found that κ_p-int_ decreased almost linearly with decreasing neck size and increasing porosity on a logarithmic scale, as shown in Fig. 4 (B and C, respectively). This finding is consistent with prior observations in nanopatterned silicon membranes (*48*). As the neck size decreases to approximately 100 nm, and the porosity increases to about 30%, the κ of the MoS_2_ PnCs drops to about 0.5 W/mK. Most notably, an ultralow effective κ of 0.1 ± 0.1 W/mK was measured in the MoS_2_ PnC with a period of 122 nm, a neck size of 29 nm, and a porosity of 40%. Compared to patterned structures in other semiconducting homogeneous materials such as Si and SiC, and other nanopatterned membranes with a similar porosity (10 to 50%), MoS_2_ exhibits notably larger values of *R*_κ_ (Fig. 3D). *R*_κ_ increased from 7.5 to ~211.8 as the porosity increased from 9 to 40%. Nanopatterned Si and SiC membranes require porosities larger than 50% to obtain reduction factors below 5 (*49*–*53*). This finding confirms that nanopatterning is an effective method to reduce the κ of MoS_2_.

The large reduction factor in MoS_2_ can also be attributed to a high degree of thermal anisotropy between the in-plane and cross-plane directions of MoS_2_, arising from the different crystalline bonds in these directions. Nanopatterning reduces the κ of materials due to diffusive phonon scattering at the hole boundaries. In isotropic materials such as Si and SiC, phonons undergo similar scattering, independent of the direction of propagation. However, in anisotropic materials, the transport of scattered phonons may be limited in certain directions (in the cross-plane direction in the case of 2D materials), making scattering more efficient, thus leading to a strong suppression of κ. The variation of *R*_κ_ with thickness is shown in fig. S9. By comparing the PnC samples with a similar period of 500 nm, an increase trend in *R*_κ_ is observed with thickness. This trend likely arises from the coupling between the phonon-surface scattering and the hole-introduced boundary scattering for thin film.

Termentzidis and colleagues have demonstrated that amorphization of the hole walls substantially influences heat transport in silicon PnCs (*54*). When considering FIB patterned MoS_2_, the presence of amorphous MoS_2_ at the hole edges also decreases κ_p-int_; however, this effect is less important in 2D materials compared to silicon due to the presence of the van der Waals interlayer gaps. Another potential explanation for the large observed *R*_κ_ is the existence of defects in the nanopatterned MoS_2_. Using FIB to pattern MoS_2_ can lead to preferential sputtering of sulfur from the samples, resulting in a change in material stoichiometry and an increased presence of sulfur vacancies (*23*, *24*). It has been shown that sulfur atoms are predominantly sputtered away from the top or bottom layers of MoS_2_ (*55*, *56*). The presence of the amorphous phase and defects, such as sulfur vacancies, can be seen in the Raman scattering spectra of the MoS_2_ PnCs (fig. S7). The absence of spatial order and long-range translational symmetry leads to a red shift of the Raman modes, a broadening of their linewidth, and a remarkable decrease in their intensities.

The figure of merit (*zT*) in thermoelectric materials exhibits a direct correlation with the square of the Seebeck coefficient and σ, while it is inversely related to κ. In the layered MoS_2_, the enhanced *zT* is expected because the electrical reduction factor (*R*_σ_) is smaller than the thermal conductivity reduction factor (*R*_κ_). Therefore, our study proposes a promising strategy to enhance the thermoelectric *zT* of layered materials by using low-porosity PnCs. The low porosity of PnCs implies that only a minor volume fraction of material is removed from the membrane, which limits any decrease in σ. Moreover, the neck size, exceeding 200 nm, notably surpasses the electron MFP, thereby reducing the electron-boundary scattering intensity in the PnCs. Effectively, this means that the thermal and electrical conductivities can be decoupled, allowing for the tunability of the thermal properties independently from the electrical ones, ultimately leading to the increase in the thermoelectric *zT*.

**Fig. 5.**
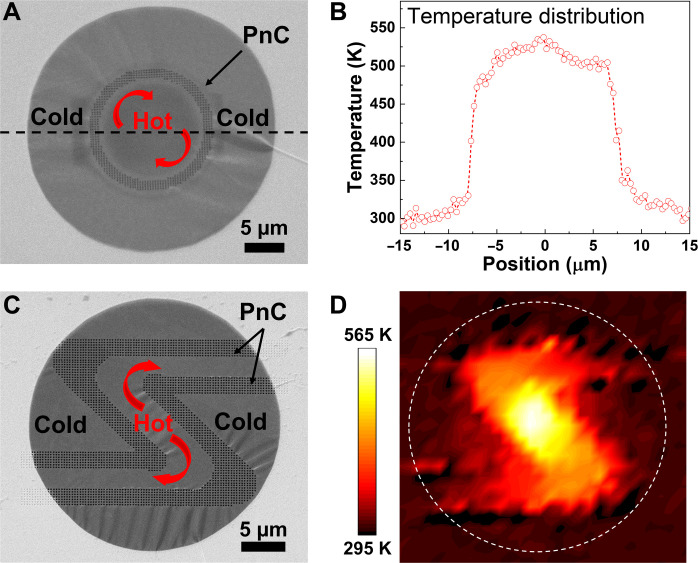
MoS_2_ thermal insulator and heat conduction channel. (**A**) SEM image of a freestanding MoS_2_ membrane patterned with a thermal insulator ring. The dashed line indicates the scan axis. (**B**) The corresponding temperature profile for the absorbed power *P* = 28.4 μW focused on the center of the sample. (**C**) SEM image of a freestanding MoS_2_ membrane with a Z-shaped heat conduction channel. (**D**) The corresponding temperature map for an absorbed power *P* = 8.5 μW focused on the center of the sample. The dashed line indicates the membrane edge.

To further advance our understanding of heat flow in patterned MoS_2_, we fabricated heat-routing structures based on freestanding, single-crystalline 10-nm-thick MoS_2_ membranes of 30-μm diameter. A heat-insulating ring with a radius *r* = 7.5 μm consisting of four lattice periods in width with *a* = 300 nm on a freestanding MoS_2_ membrane is shown in Fig. 5A and fig. S11. The heating laser was focused on the center of the membrane to create a hotspot. The temperature profile for the absorbed heating power *P* = 28.4 μW was recorded along the dashed line in Fig. 5B. The center of the membrane, i.e., inside the ring, maintained a temperature of approximately 500 K. A sharp drop of the temperature down to 330 K was recorded between the inner and outer parts of the ring, indicating that the ring blocked most of the heat flow from the center to the edges of the membrane. The temperature of the outer part of the MoS_2_ was almost unaffected and maintained a temperature between 300 and 330 K since it was thermally anchored to the supporting metal-coated frame. All measurements were performed in a vacuum to avoid convection or heat transfer to air. Therefore, no other dissipation channels were available, and the only path for the heat to flow was through the patterned area. The corresponding thermal resistance *R*_th_ of the patterned region can be calculated from *Q* = Δ*T/R*_th_, where *Q* is the heat flux and Δ*T* is the temperature drop. The calculated *R*_th_ is 4 × 10^−6^ m^2^ K/W, which corresponds to a thermal boundary conductance *G* = 1/*R*_th_ = 0.25 MW/m^2^ K and κ_p-int_ below 1 W/mK.

We extended this experiment by fabricating a Z-shaped heat guiding channel (*a* = 300 nm; patterned area width, 2.1 μm; channel width, 3.6 μm) on the MoS_2_ membrane (Fig. 5C and fig. S12). The heating laser was focused on the center of the membrane, creating a hotspot with a temperature of 560 K. Figure 5D shows the temperature distribution on the sample. The temperature inside the channel decreased from the hotspot toward the heat sink at the membrane edges. Thus, the lateral heat flow was delimited by the patterned area, and the area outside the channel was protected from high temperatures. The temperature outside the channel was close to that of the heat sink, at about 300 K. Such directional heat flow is not possible using existing heat spreading materials, such as copper or sintered silver pastes, where thermal transport is isotropic. Compared to existing 2D materials, the ultralow value of κ for nanopatterned MoS_2_ is comparable to its polycrystalline counterpart in the nanometer-scale grain size limit, which is in the range of 0.5 to 2 W/mK (*21*). To the best of our knowledge, this is the first attempt to create thermal interfaces in the in-plane direction in 2D materials that exhibit such high *R*_th_ due to such an effect. Moreover, *R*_th_ scales linearly with the width of the nanopatterned region, which provides another degree of freedom in the design of the heat-routing structures based on PnCs. Our device-like structures perform well even when the temperature of the hotspot is much higher than the hotspot temperature in a real electronic circuit, which is on the order of 400 K (*57*).

In summary, we have fabricated suspended crystalline MoS_2_ membranes of various thicknesses and applied advanced FIB-based nanopatterning techniques to develop PnCs in these membranes. Our comprehensive investigation into the thermal conductivity of MoS_2_ before and after nanopatterning encompasses a range of experimental and theoretical methodologies, including 1LRT, 2LRT, MD simulation, MFP reconstruction method, and COMSOL simulations. The nanopatterning technique has proven to be highly effective at enabling efficient thermal management. We achieved an unprecedentedly low thermal conductivity value of 0.1 ± 0.1 W/mK for the layered crystalline PnC structure. Our findings demonstrate the exceptional efficiency of these layered PnCs in reducing κ, surpassing the capabilities of Si and SiC PnCs with comparable periodicity by approximately 100-fold. Furthermore, we have realized MoS_2_-based thermal routing nanostructures for thermal isolation and heat guidance in prespecified directions. Our strategy can be readily extended to other layered materials, such as graphene or hBN, which have high thermal conductivities that make them interesting candidates for heat spreading applications. These findings enable innovative strategies for thermal management in future 3D IC electronics and thermoelectric devices containing 2D layered materials. By using nanopatterned 2D layered materials to reduce thermal conductivity while maintaining electrical conductivity, substantial improvement in both thermal management efficiency for electronics and waste heat conversion efficiency for thermoelectric devices can be achieved.

## MATERIALS AND METHODS

### Sample fabrication

An Au/Ti (95 nm/5 nm) layer was deposited on a SiNx TEM grid substrate (Norcada Inc., Canada) by e-beam metal deposition. A 2-mm thick polydimethylsiloxane (PDMS) film was created using a 10:1 ratio of silicon base to curing agent (Sylgard 184, Dow Corning, USA) and cured at room temperature for 24 hours. The PDMS film was used to mechanically exfoliate MoS_2_ membranes from bulk MoS_2_ crystals (Graphene Supermarket, USA), and the MoS_2_ membranes were dry transferred onto the Au/Ti-coated substrate, leaving a freestanding MoS_2_ membrane.

An FIB (Zeiss 1560XB Cross Beam, Germany) was used to mill periodic holes into the MoS_2_ membrane with a beam current of 2 pA, a voltage of 30 kV, and an etch time of 10 ms. An unetched center area of diameter ~5 μm was left on each membrane to be used as the heating island.

### Thermal conductivity measurement

#### Two-laser Raman thermometry

As the frequency of a Raman mode depends on the material temperature, it can be used to probe the material temperature. The A_1g_ peak position of the MoS_2_ Raman spectrum was used to probe the temperature of MoS_2_ membranes in this work, as its peak intensity is much stronger than that of the E^1^_2g_ peak.

A probe laser (532 nm, Cobolt) and a heating laser (405 nm, Cobolt) were focused on the center of a freestanding membrane from the top side and bottom side, respectively. The heating laser and the freestanding membrane were fixed on a motorized stage (Märzhäuser), which sets their relative position and prevents a change in the position of the heating laser during measurement to ensure a stable and uncontacted heating source. The probe laser was coupled to the Raman spectrometer (Horiba T64000) and scanned at various points on the sample. The probe laser spot size was 1.2 μm. The laser power absorbed by the membranes was measured in situ using a configuration described elsewhere (*20*, *27*).

All samples were measured in a temperature-controlled vacuum chamber at a pressure of ~3 × 10^−3^ mtorr (Linkam). The gold layer also acted as a heat sink to ensure that the MoS_2_ temperature in the supported area is the same as that of the vacuum chamber. To calculate the thermal conductivity, a temperature distribution around the heating source on the freestanding membrane was required.

The 2LRT experiment consists of two consecutive scans: (i) no heating applied (“baseline”)—this measurement also helps to assess the sample quality (strain, contamination, etc.); (ii) heating applied using a 405-nm wavelength laser coupled from below the sample. The A_1g_ frequency difference between the background scan and heating scan was divided by the A_1g_ peak’s temperature coefficient of each sample and converted to the temperature (fig. S1 and table S1). The profiles of regions near the heat sink could be well fitted by the constant κ (Fig. 2E) where the slope of the fitting lines corresponds to the *dT*/*d*(*lnr*). The spectra are collected every 0.5 μm using a Märzhäuser stage with a Tango controller, which provides a repeatability <1 μm (bidirectional) and a resolution of 0.01 μm (smallest step size).

#### One-laser Raman thermometry

A heating-probe laser (532 nm, Cobolt) was used to heat the samples at the center as well as collect the corresponding Raman spectra. All the measurements were performed in a temperature-controlled vacuum chamber (Linkam) and the laser power absorbed by the samples was measured in situ using the configuration described elsewhere (*21*).

## Supplementary Materials

### This PDF file includes:

Figs. S1 to S14 Tables S1 to S4 References
