# Supplementary material for: MoS2 phononic crystals for advanced thermal management
Source: Sci Adv. 2024 Mar 29;10(13):eadm8825. doi: 10.1126/sciadv.adm8825 (PMC10980264; doi:10.1126/sciadv.adm8825)
Supplement: Supplementary file 1 — Figs. S1 to S14 Tables S1 to S4 References [file sciadv.adm8825_sm.pdf]

Science Advances
AAAS

Supplementary Materials for

MoS_2 phononic crystals for advanced thermal management

Peng Xiao *et al.*

Corresponding author: Peng Xiao, peng.xiao@icn2.cat; Alexandros El Sachat, a.elsachat@inn.demokritos.gr; Marianna Sledzinska, marianna.sledzinska@icn2.cat

Sci. Adv. **10**, eadm8825 (2024)
DOI: 10.1126/sciadv.adm8825

This PDF file includes:

Figs. S1 to S14
Tables S1 to S4
References

1. Sample preparation and characterization

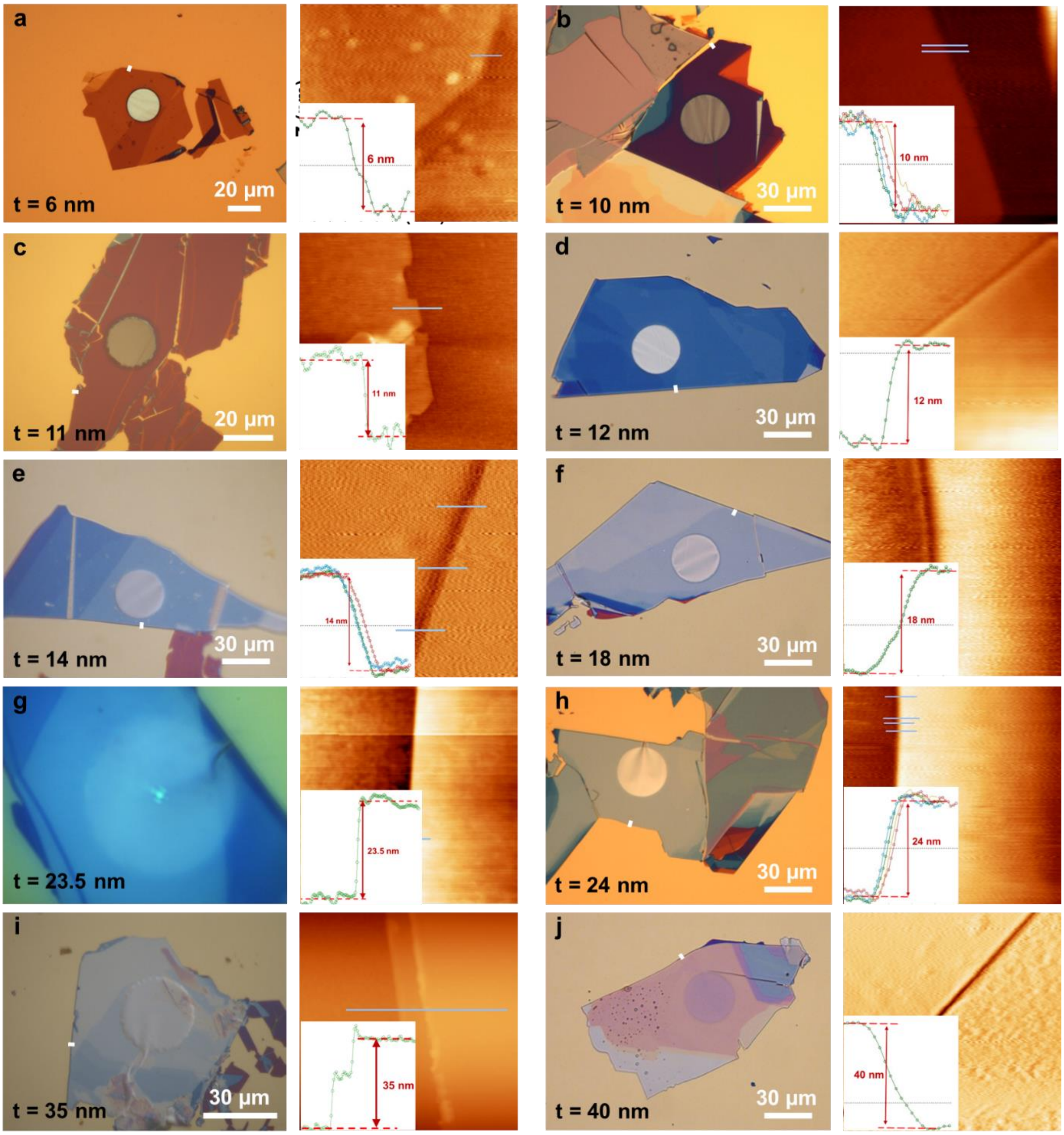

Fig. S1. Optical images, and atomic force microscopy profiles of the MoS_2 samples (a-j).

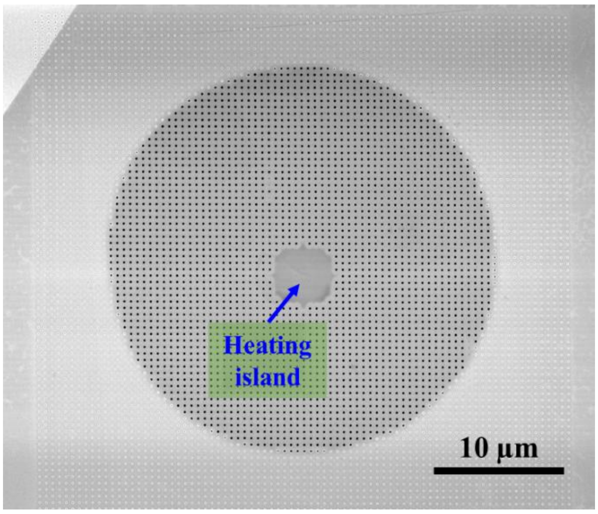

Fig. S2. SEM image of a typical MoS_2 PnC membrane. The unpatterned central region was used as heating island for the thermal measurements.

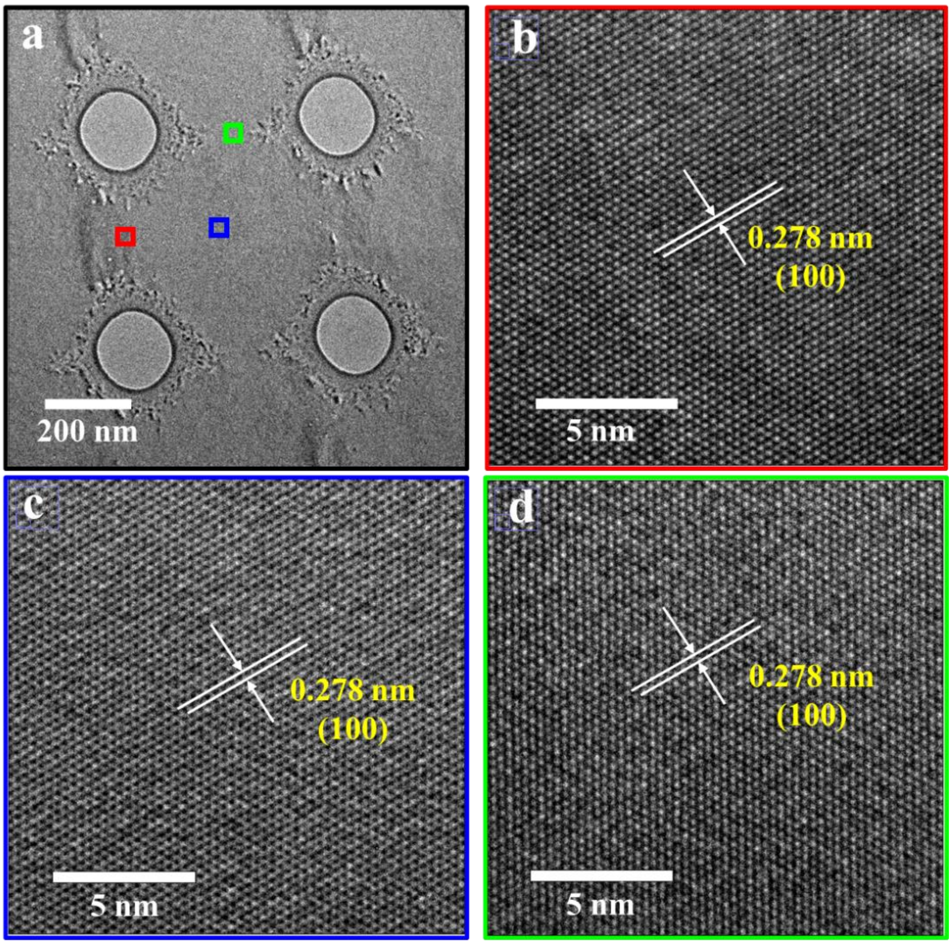

Fig. S3. TEM images of the patterned 4.5 nm thick MoS_2 membrane. (a) shows the morphology of a typical PnC. (b), (c), and (d) respectively show the corresponding regions in the red, blue, and green boxes in (a). The results indicate that the areas away from the holes are crystalline, and the lattice dimensions remain unchanged.

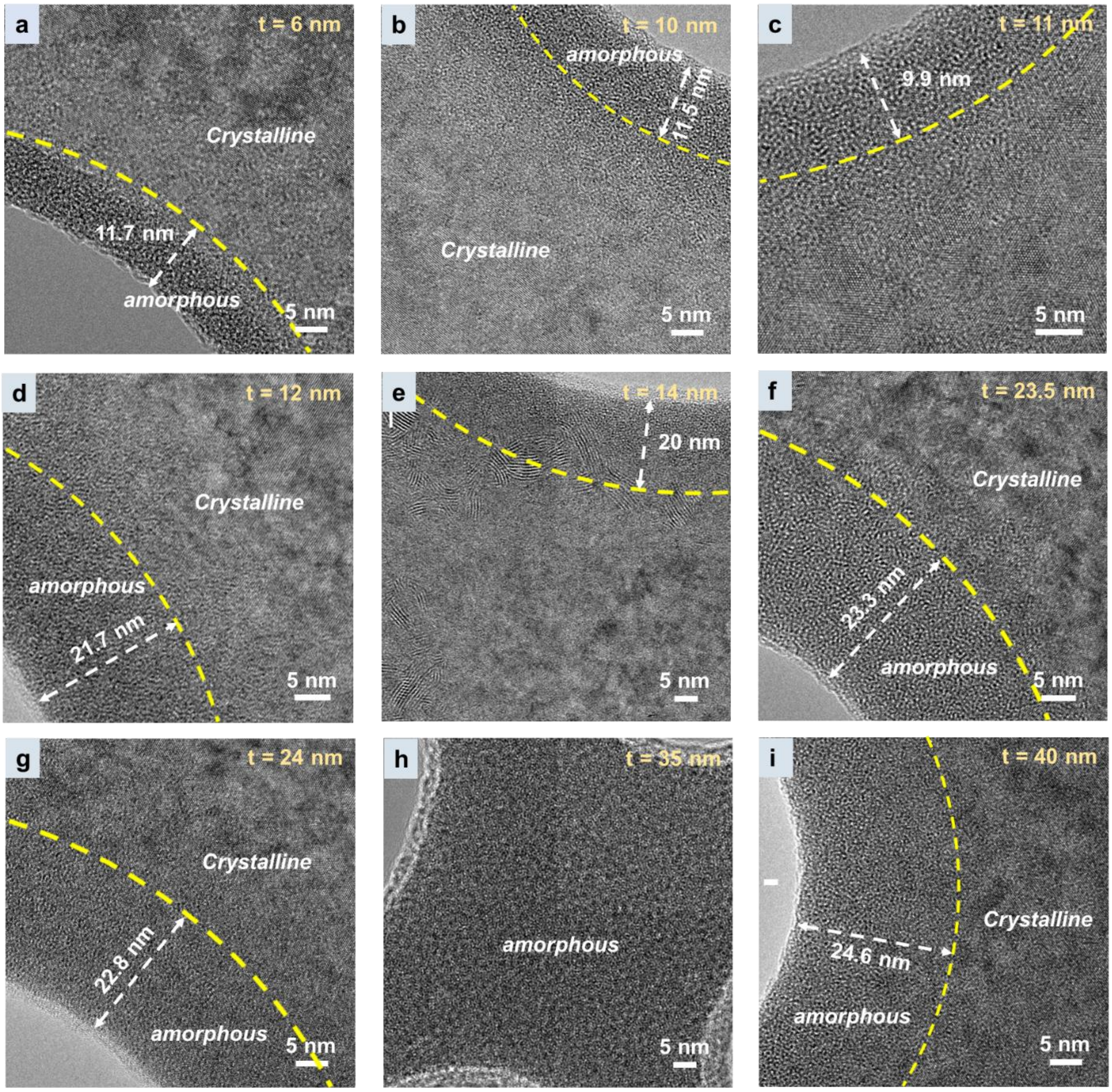

Fig. S4. TEM images of the patterned MoS_2 samples (a-i).

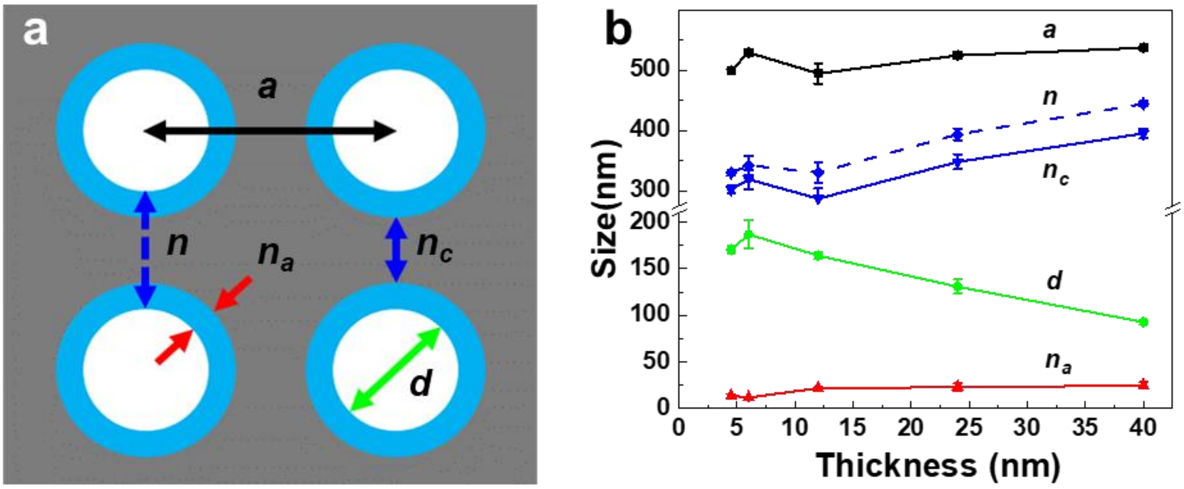

Fig. S5. Thickness-dependent dimensions of PnCs. (a) Schematic diagram of the MoS_2 PnCs, $\boldsymbol{a}$ is the period, $\boldsymbol{n}$ is the neck size, $\boldsymbol{n_c}$ is the neck size without the amorphous region, $\boldsymbol{d}$ is the hole diameter, and $\boldsymbol{n_a}$ is the size of the amorphous region. (b) Variation of each parameter shown in (a) with respect to membrane thickness while keeping FIB processing conditions at 30kV and 2pA.

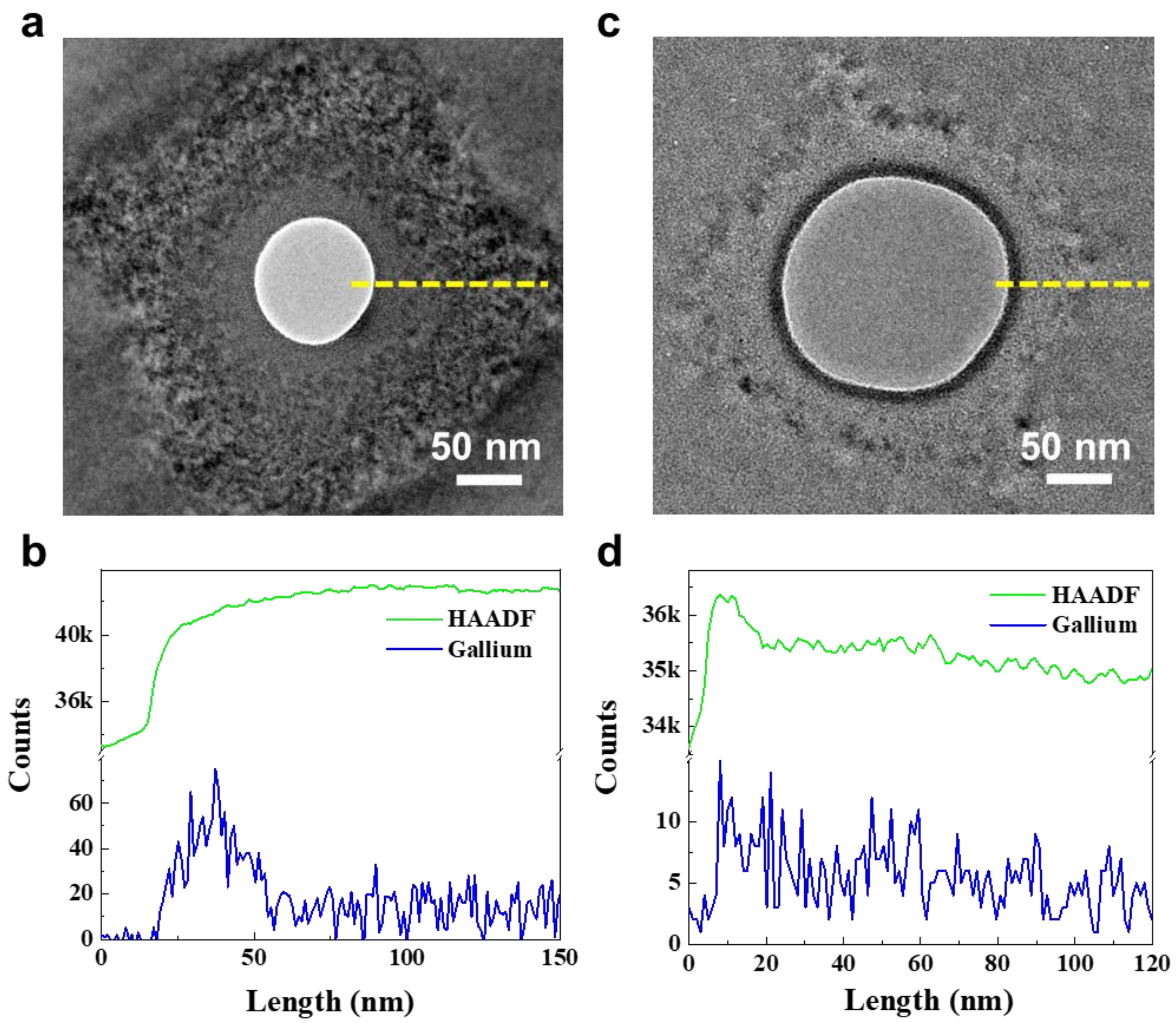

Fig. S6. STEM images (a and c) and high-angle annular dark-field (HAADF) imaging profiles and gallium atom trace (b and d) for MoS_2 patterned samples with thicknesses of 40 nm (a and b) and 4.5 nm (c and d).

2. Thermal conductivity measurements

Temperature-dependent thermal conductivity: The measurements were performed in a temperature-controlled vacuum chamber (Linkam) where the heatsink temperature varied from 123 K to 473 K. The samples were characterized using the 1LRT.

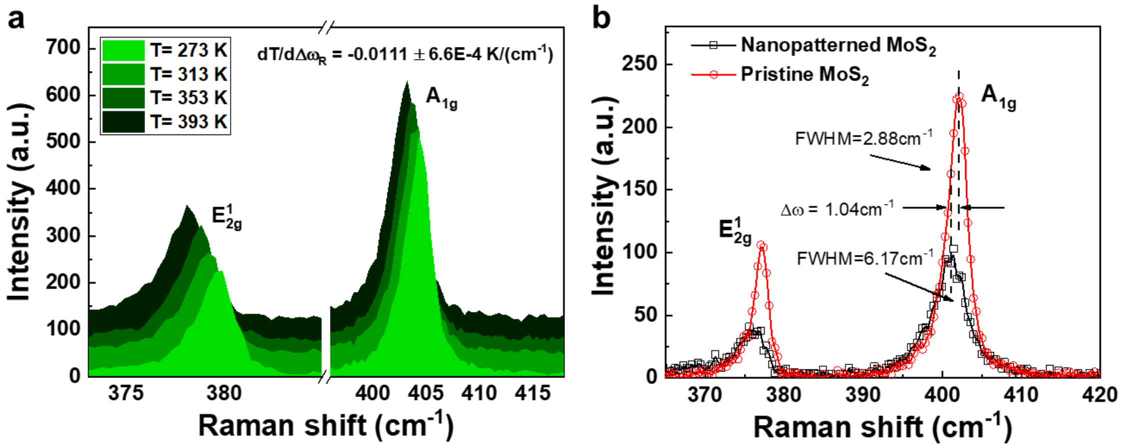

Fig. S7. Raman spectra of MoS_2. (a) Temperature-dependent Raman spectra of the MoS_2. (b) Raman spectra of the pristine and nanopatterned MoS_2.

$dT/d\Delta\omega_R$ (K/cm^{-1})		Measured area (Supported/suspended)			
		MoS_2 A_{1g}- (Supported)	MoS_2 A_{1g}- (Suspended)	MoS_2 E^1_{2g}- (Supported)	MoS_2 E^1_{2g}- (Suspended)
Thickness (nm)	**4.5**	-0.0112 ± 0.00090	-0.0111 ± 0.00066	-0.0127 ± 0.00067	-0.0125 ± 0.00053
	**6**	-0.0088 ± 0.00063	-0.0102 ± 0.00027	-0.0104 ± 0.00011	-0.0117 ± 0.00007
	**10**	-0.0085 ± 0.00085	-0.0090 ± 0.00019	-0.0010 ± 0.00130	-0.0101 ± 0.00063
	**12**	-0.0099 ± 0.00039	-0.0097 ± 0.00061	-0.0108 ± 0.00032	-0.0114 ± 0.00091
	**14**	-0.0093 ± 0.00270	-0.0108 ± 0.00260	-0.0133 ± 0.00043	-0.0146 ± 0.00170
	**18**	-0.0100 ± 0.00031	-0.0100 ± 0.00022	-0.0117 ± 0.00061	-0.0109 ± 0.00035
	**24**	-0.0096 ± 0.00100	-0.0085 ± 0.00047	-0.0090 ± 0.00220	-0.0121 ± 0.00170
	**40**	-0.0107 ± 0.00013	-0.0107 ± 0.00012	-0.0120 ± 0.00046	-0.0124 ± 0.00047

Table S1. Temperature dependence of the MoS_2 A_{1g} and E_{2g} Raman peak positions for the samples used in this study.

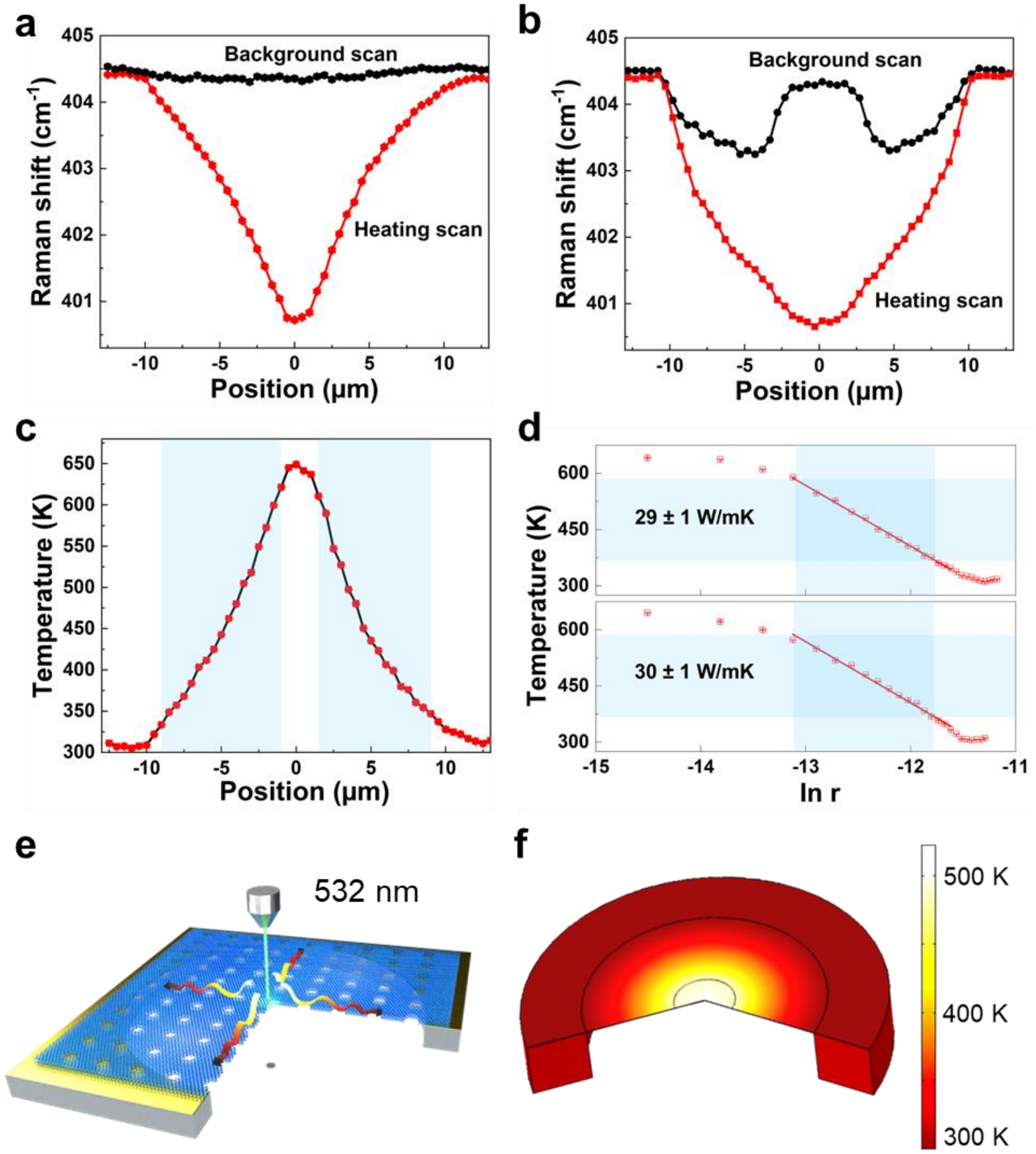

Fig. S8. Thermal conductivity measurements of MoS_2 PnCs. (a-d) Thermal conductivity of the 4.5 nm-thick, free-standing pristine and nanopatterned MoS_2 membranes measured by 2LRT. (a) *Background and Heating scans* of pristine MoS_2 suspended film. These scans were measured using a probe laser (532 nm) with an incident power P_{probe} ~10 µW, and a heating laser (405 nm) with an absorbed power of P_{pump} = 137.5 µW. The heating laser was focused onto the center of the sample (r = 0 µm) for *the heating scans*. (b) *Background and Heating scans* of MoS_2 PnCs suspended film. These scans were measured using a probe laser with an incident power of P_{probe} ~6.8 µW, and a heating laser with an absorbed power of P_{pump} = 15.8 µW. (c) Temperature profile on the sample, extracted from (a), and (d) shows the corresponding

temperature profile as a function of ln(*r*). The solid line represents a linear fit of the experimental points. (e) Schematic diagram of the 1LRT-based thermal conductivity measurement of MoS_2 PnCs, and (f) COMSOL model used in the thermal conductivity calculation of the 1LRT.

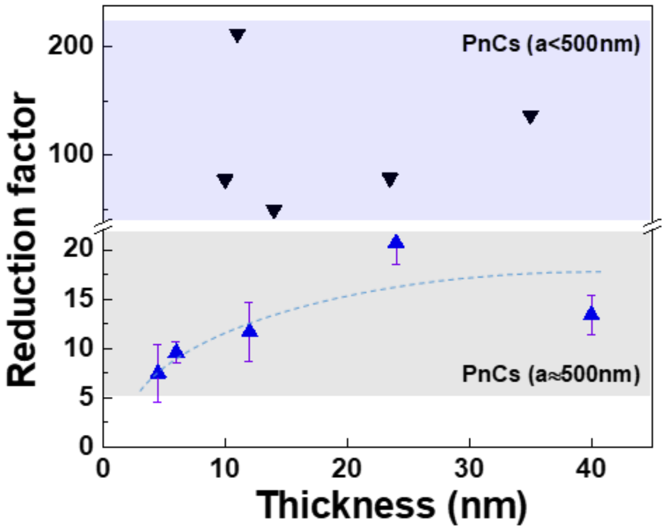

Fig. S9. Thermal conductivity reduction factor of nanopatterned MoS_2 as a function of the membrane thickness.

3. Electrical conductivity measurement

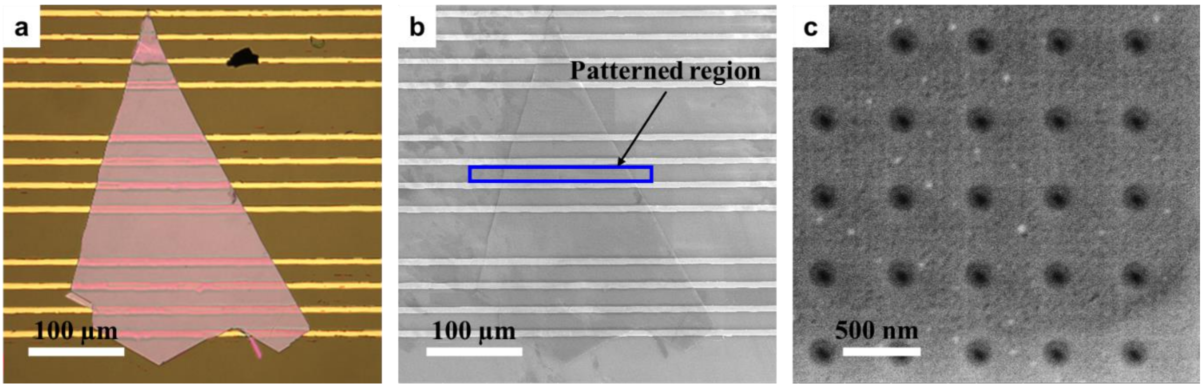

Fig. S10. Electrical conductivity measurement of nanopatterned MoS_2. (a) Optical image of a typical MoS_2 device. (b) and (c) SEM images of (a) and PnC in the patterned region in (b), respectively.

	Thickness (nm)	Period (nm)	$\sigma_{pristine}$ (S/m)	σ_{pnc} (S/m)	$R\sigma$
MoS_2-1	30	500	~182	~38	4.8
MoS_2-2	118	500	~118	~57	2.1

Table S2. Electrical conductivity of FIB-patterned layered MoS_2. $R\sigma = \sigma_{pristine} / \sigma_{pnc}$.

4. MoS_2 thermal insulator and heat conduction channel

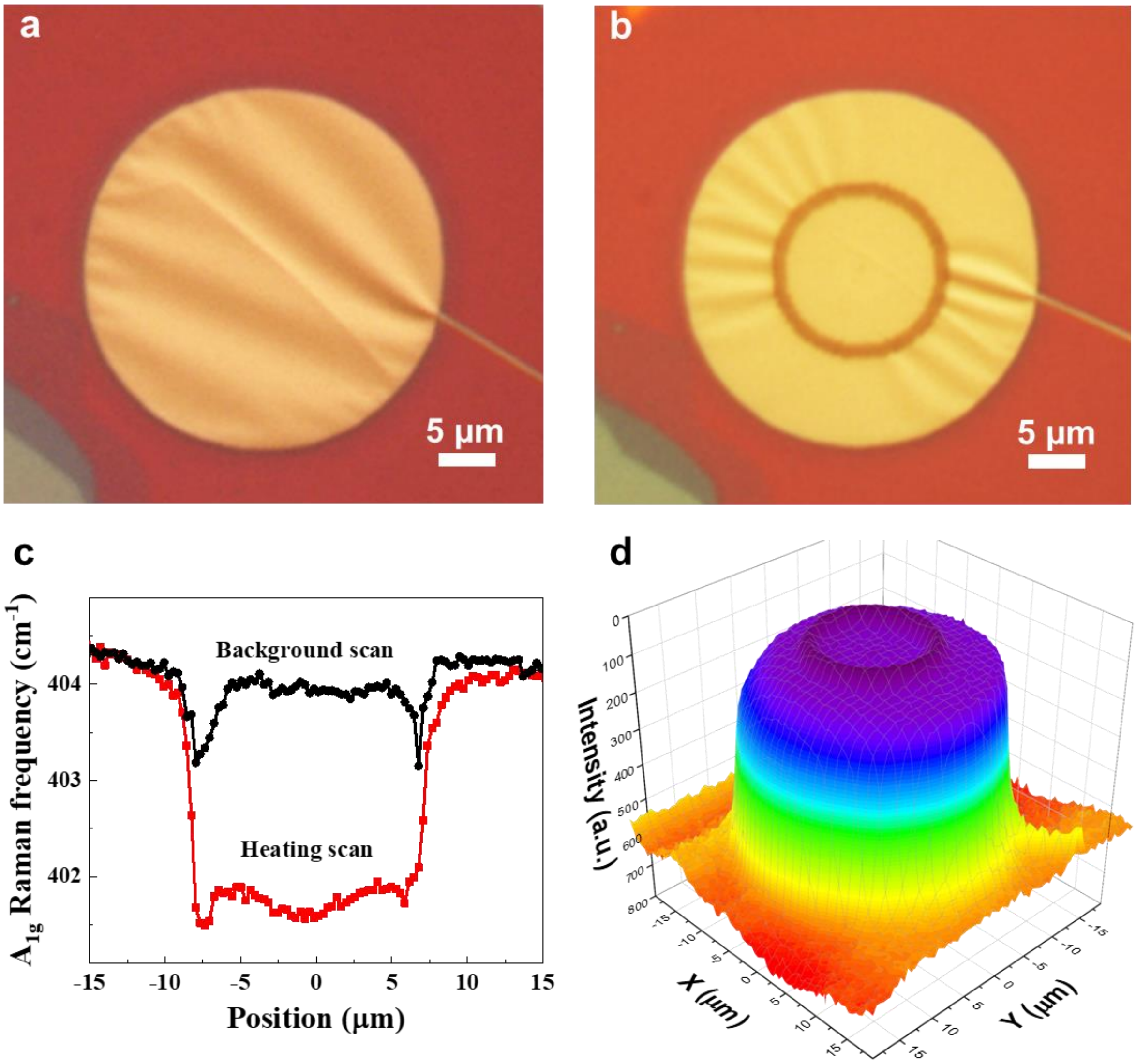

Fig. S11. Optical images of (a) pristine and (b) nanopatterned MoS_2. (c) A_{1g} Raman frequency with and without heating as a function of position. (d) A_{1g} Raman peak intensity as a function of position.

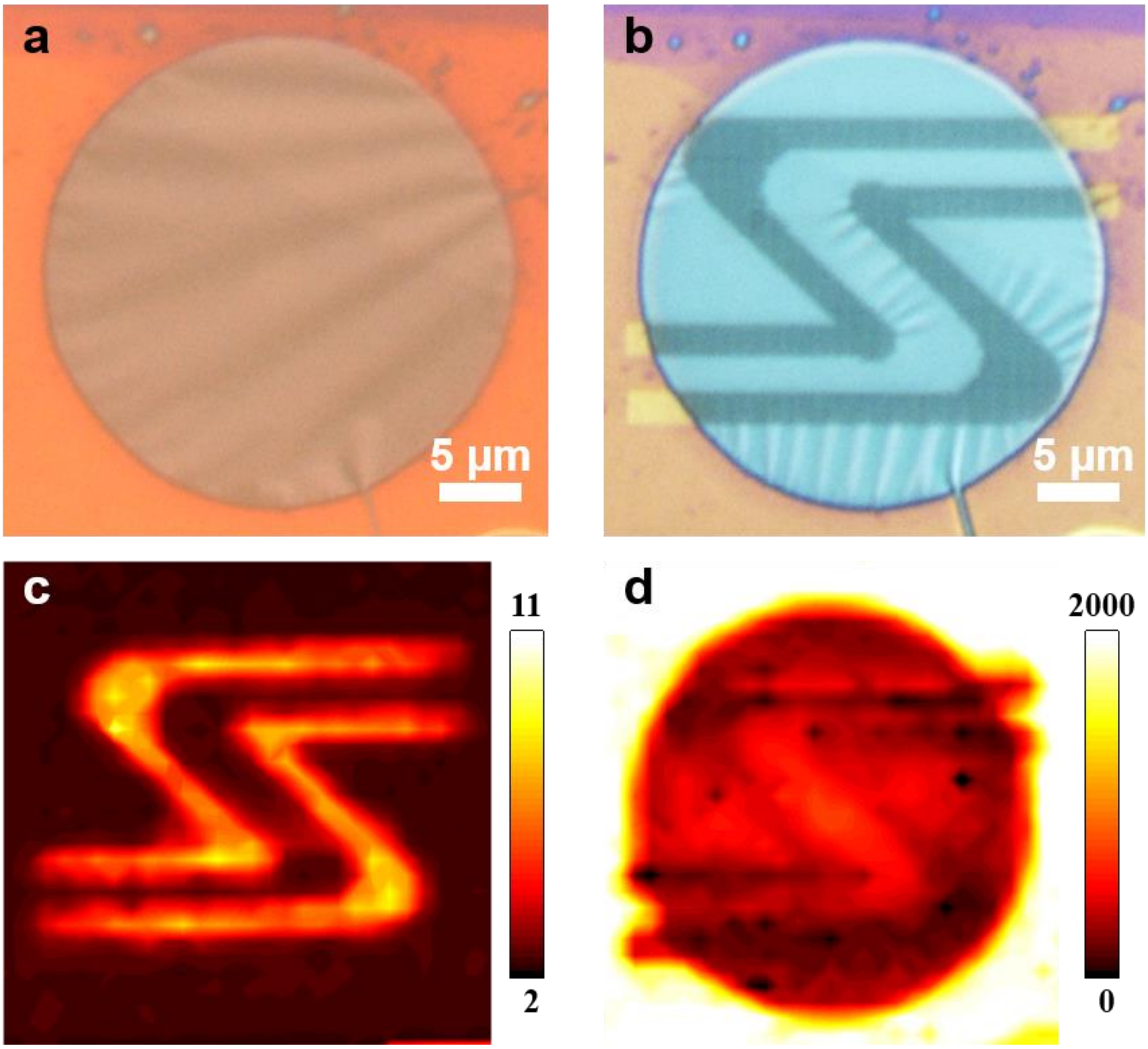

Fig. S12. Optical images of (a) pristine and (b) nanopatterned MoS_2. (c) A_{1g} Raman peak FWHM. (d) A_{1g} Raman peak intensity as a function of position.

5. MD simulations

Concerning the dependence of the thermal conductivity with the layer thickness, three sizes have been investigated; 4 nm (7 tri-layers), 10 nm (17 tri-layers) and a bulk system at 300 K as presented in Figure 3. It should be noted that even for 10 nm thickness, the bulk conductivity has not been reached in contrast to ref (*58*) which claims that the thermal conductivity has already reached that of bulk values from 3 tri-layers onwards (about 1.8 nm). The discrepancy is based on the fact that in the present study, the well-established EMD method is used extensively (averaging the results of 10 different cases having random initial distribution of atomic velocities) while in ref (*58*) the authors have used the homogeneous nonequilibrium MD (HNEMD) method (*59, 60*) in the recently developed form, which is constructed in order for the HNEMD method to become efficient for general many-body potentials, including the REBO potential (*61*). The current outcome using the EMD method supports the validity of the aforementioned methodology in combination with the selected interatomic potential owing to the agreement with the experimental results.

Thickness (nm)	Temperature (K)	Thermal conductivity (W/mK)	
		in-plane	RMSE
4	300	8.9	2.7
10	130	43.8	52.4
10	200	34.5	14.9
10	300	23.9	7.3
10	400	16.7	8.9
10	500	9.5	2.9
bulk	300	38.5	9.8

Table S3. Thickness dependence of thermal conductivity of MoS_2 membranes.

For the main focus of our work, we scaled down the porous, thin film for computational optimization, to study its thermal conductivity. Table S3 shows information about the original pristine system and the scaled down system that we studied. To make sure the scaled down system is acceptable for our simulation, we took into account the phonon means free path (PMFP). The crystalline surface of the system should be large enough, so it does not restrict the phononic vibrations. Therefore, the smallest path between two consecutive holes is equal to the periodicity minus the hole diameter. This smallest path evaluates to 62.4 nm which is larger than the PMFP = 41 nm and hence, the scale down of the system does not restrict the thermal conductivity in any way. The in-plane thermal conductivity was calculated to be equal to 5.2 W/mK which compares well with our experimental results.

	Thickness (nm)	Hole dimeter (nm)	Periodicity (nm)	Amorphous size (nm)	Number of atoms	x axis (nm)	y axis (nm)
Original	4	177	489	13.5	58.958.892	489	489
Scaled down	4	35.4	97.8	3	2.037.703	97.32	97.14

Table S4. Values of the sample parameters and the scaled down values used in the MD simulation.

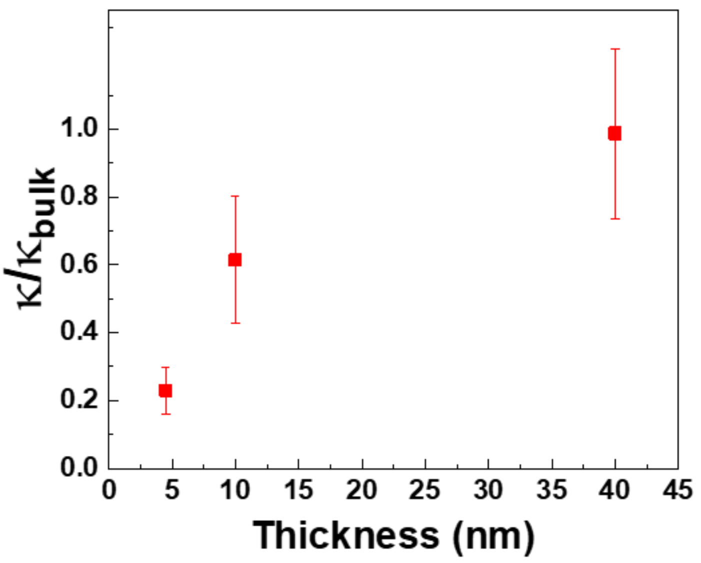

Fig. S13. EMD simulations (a) Three atomistic configurations: bulk, 10 nm, and 4 nm thick. (b) Normalized thermal conductivity as a function of thickness.

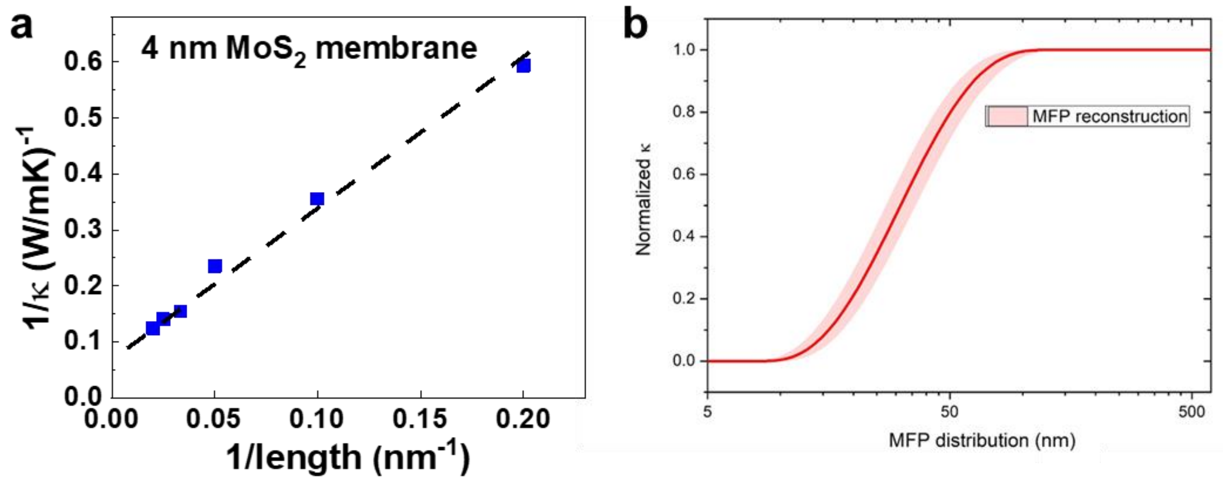

Fig. S14. Normalized accumulated thermal conductivity as a function of the phonon MFP.

REFERENCES AND NOTES

1. H. S. Lee, K. Chakrabarty, Test challenges for 3D integrated circuits. *IEEE Design Test of Computer* **26**, 26–35 (2009).

2. Y. Cui, M. Li, Y. Hu, Emerging interface materials for electronics thermal management: Experiments, modeling, and new opportunities. *J. Mater. Chem. C* **8**, 10568–10586 (2020).

3. Y. Fu, J. Hansson, Y. Liu, S. Chen, A. Zehri, M. K. Samani, N. Wang, Y. Ni, Y. Zhang, Z.-B. Zhang, Q. Wang, M. Li, H. Lu, M. Sledzinska, C. M. S. Torres, S. Volz, A. A. Balandin, X. Xu, J. Liu, Graphene related materials for thermal management. *2D Mater.* **7**, 012001 (2019).

4. S. Lohrasbi, R. Hammer, W. Essl, G. Reiss, S. Defregger, W. Sanz, A comprehensive review on the core thermal management improvement concepts in power electronics. *IEEE Access* **8**, 166880–166906 (2020).

5. H. Song, J. Liu, B. Liu, J. Wu, H.-M. Cheng, F. Kang, Two-dimensional materials for thermal management applications. *Joule* **2**, 442–463 (2018).

6. A. Licht, N. Pfiester, D. DeMeo, J. Chivers, T. E. Vandervelde, A review of advances in thermophotovoltaics for power generation and waste heat harvesting. *MRS Advances* **4**, 2271–2282 (2019).

7. E. Chavez-Angel, P. Tsipas, P. Xiao, M. T. Ahmadi, A. H. S. Daaoub, H. Sadeghi, C. M. Sotomayor Torres, A. Dimoulas, A. E. Sachat, Engineering heat transport across epitaxial lattice-mismatched van der Waals heterointerfaces. *Nano Lett.* **23**, 6883–6891 (2023).

8. P. Yuan, R. Wang, T. Wang, X. Wang, Y. Xie, Nonmonotonic thickness-dependence of in-plane thermal conductivity of few-layered MoS_2: 2.4 to 37.8 nm. *Phys. Chem. Chem. Phys.* **20**, 25752–25761 (2018).

9. M. Yarali, X. Wu, T. Gupta, D. Ghoshal, L. Xie, Z. Zhu, H. Brahmi, J. Bao, S. Chen, T. Luo, N. Koratkar, A. Mavrokefalos, Effects of defects on the temperature-dependent thermal conductivity of

suspended monolayer molybdenum disulfide grown by chemical vapor deposition. *Adv. Funct. Mater.* **27**, 1704357 (2017).

10. A. Aiyiti, S. Hu, C. Wang, Q. Xi, Z. Cheng, M. Xia, Y. Ma, J. Wu, J. Guo, Q. Wang, J. Zhou, J. Chen, X. Xu, B. Li, Thermal conductivity of suspended few-layer MoS_2. *Nanoscale* **10**, 2727–2734 (2018).

11. J. J. Bae, H. Y. Jeong, G. H. Han, J. Kim, H. Kim, M. S. Kim, B. H. Moon, S. C. Lim, Y. H. Lee, Thickness-dependent in-plane thermal conductivity of suspended MoS_2 grown by chemical vapor deposition. *Nanoscale* **9**, 2541–2547 (2017).

12. X. Gu, B. Li, R. Yang, Layer thickness-dependent phonon properties and thermal conductivity of MoS_2. *J. Appl. Phys.* **119**, 085106 (2016).

13. A. Sood, F. Xiong, S. Chen, R. Cheaito, F. Lian, M. Asheghi, Y. Cui, D. Donadio, K. E. Goodson, E. Pop, Quasi-ballistic thermal transport across MoS_2 thin films. *Nano Lett.* **19**, 2434–2442 (2019).

14. J. Liu, G.-M. Choi, D. G. Cahill, Measurement of the anisotropic thermal conductivity of molybdenum disulfide by the time-resolved magneto-optic Kerr effect. *J. Appl. Phys.* **116**, 233107 (2014).

15. A. Islam, A. van den Akker, P. X.-L. Feng, Anisotropic thermal conductivity of suspended black phosphorus probed by opto-thermomechanical resonance spectromicroscopy. *Nano Lett.* **18**, 7683–7691 (2018).

16. B. Kurşun, M. Sivrioğlu, Heat transfer enhancement using U-shaped flow routing plates in cooling printed circuit boards. *J. Braz. Soc. Mech. Sci. Eng.* **40**, 13 (2018).

17. J. Song, L. Lu, B. Li, B. Zhang, R. Hu, X. Zhou, Q. Cheng, Thermal routing via near-field radiative heat transfer. *Int. J. Heat Mass Transf.* **150**, 119346 (2020).

18. M. Buscema, M. Barkelid, V. Zwiller, H. S. J. van der Zant, G. A. Steele, A. Castellanos-Gomez, Large and tunable photothermoelectric effect in single-layer MoS_2. *Nano Lett.* **13**, 358–363 (2013).

19. R. Mansfield, S. A. Salam, Electrical properties of molybdenite. *Proc. Phys. Soc. B* **66**, 377–385 (1953).

20. M. Sledzinska, B. Graczykowski, M. Placidi, D. S. Reig, A. E. Sachat, J. S. Reparaz, F. Alzina, B. Mortazavi, R. Quey, L. Colombo, S. Roche, C. M. S. Torres, Thermal conductivity of MoS_2 polycrystalline nanomembranes. *2D Mater.* **3**, 035016 (2016).

21. M. Sledzinska, R. Quey, B. Mortazavi, B. Graczykowski, M. Placidi, D. S. Reig, D. Navarro-Urrios, F. Alzina, L. Colombo, S. Roche, C. M. S. Torres, Record low thermal conductivity of polycrystalline MoS_2 films: Tuning the thermal conductivity by grain orientation. *ACS Appl. Mater. Interfaces* **9**, 37905–37911 (2017).

22. M. G. Stanford, P. D. Rack, D. Jariwala, Emerging nanofabrication and quantum confinement techniques for 2D materials beyond graphene. *npj 2D Mat. Appl.* **2**, 1–15 (2018).

23. D. S. Fox, Y. Zhou, P. Maguire, A. O'Neill, C. Ó'Coileáin, R. Gatensby, A. M. Glushenkov, T. Tao, G. S. Duesberg, I. V. Shvets, M. Abid, M. Abid, H.-C. Wu, Y. Chen, J. N. Coleman, J. F. Donegan, H. Zhang, Nanopatterning and electrical tuning of MoS_2 layers with a subnanometer helium ion beam. *Nano Lett.* **15**, 5307–5313 (2015).

24. R. Mupparapu, M. Steinert, A. George, Z. Tang, A. Turchanin, T. Pertsch, I. Staude, Facile resist-free nanopatterning of monolayers of MoS_2 by focused ion-beam milling. *Adv. Mater. Interfaces* **7**, 2000858 (2020).

25. F. Liu, M. Muruganathan, Y. Feng, S. Ogawa, Y. Morita, C. Liu, J. Guo, M. Schmidt, H. Mizuta, Thermal rectification on asymmetric suspended graphene nanomesh devices. *Nano Futures* **5**, 045002 (2021).

26. M. Sledzinska, B. Graczykowski, J. Maire, E. Chavez-Angel, C. M. Sotomayor-Torres, F. Alzina, 2D phononic crystals: Progress and prospects in hypersound and thermal transport engineering. *Adv. Funct. Mater.* **30**, 1904434 (2020).

27. B. Graczykowski, A. El Sachat, J. S. Reparaz, M. Sledzinska, M. R. Wagner, E. Chavez-Angel, Y. Wu, S. Volz, Y. Wu, F. Alzina, C. M. Sotomayor Torres, Thermal conductivity and air-mediated losses in periodic porous silicon membranes at high temperatures. *Nat. Commun.* **8**, 415 (2017).

28. Y. Cai, J. Lan, G. Zhang, Y.-W. Zhang, Lattice vibrational modes and phonon thermal conductivity of monolayer MoS_2. *Phys. Rev. B* **89**, 035438 (2014).

29. M. Zulfiqar, Y. Zhao, G. Li, Z. Li, J. Ni, Intrinsic thermal conductivities of monolayer transition metal dichalcogenides MX_2 (M = Mo, W; X = S, Se, Te). *Sci. Rep.* **9**, 4571 (2019).

30. S. Xiao, P. Xiao, X. Zhang, D. Yan, X. Gu, F. Qin, Z. Ni, Z. J. Han, K. Ostrikov, Atomic-layer soft plasma etching of MoS_2. *Sci. Rep.* **6**, 19945 (2016).

31. P. Xiao, E. Chavez-Angel, S. Chaitoglou, M. Sledzinska, A. Dimoulas, C. M. Sotomayor Torres, A. El Sachat, Anisotropic thermal conductivity of crystalline layered $SnSe_2$. *Nano Lett.* **21**, 9172–9179 (2021).

32. A. Arrighi, E. D. Corro, D. N. Urrios, M. V. Costache, J. F. F. Sierra, K. Watanabe, T. Taniguchi, J. A. Garrido, S. O. Valenzuela, C. M. S. Torres, M. Sledzinska, Heat dissipation in few-layer MoS_2 and MoS_2/hBN heterostructure. *2D Mater.* **9**, 015005 (2021).

33. A. El Sachat, F. Alzina, C. M. Sotomayor Torres, E. Chavez-Angel, Heat transport control and thermal characterization of low-dimensional materials: A review. *Nanomaterials* **11**, 175 (2021).

34. J. S. Reparaz, E. Chavez-Angel, M. R. Wagner, B. Graczykowski, J. Gomis-Bresco, F. Alzina, C. M. Sotomayor Torres, A novel contactless technique for thermal field mapping and thermal conductivity determination: Two-laser raman thermometry. *Rev. Sci. Instrum.* **85**, 034901 (2014).

35. R. C. Ng, A. El Sachat, F. Cespedes, M. Poblet, G. Madiot, J. Jaramillo-Fernandez, O. Florez, P. Xiao, M. Sledzinska, C. M. Sotomayor-Torres, E. Chavez-Angel, Excitation and detection of acoustic phonons in nanoscale systems. *Nanoscale* **14**, 13428–13451 (2022).

36. Z. Hashin, S. Shtrikman, A variational approach to the theory of the effective magnetic permeability of multiphase materials. *J. Appl. Phys.* **33**, 3125–3131 (1962).

37. K. Termentzidis, *Nanostructured Semiconductors Amorphization and Thermal Properties* (Jenny Stanford Publishing, 2017).

38. S. Plimpton, Fast parallel algorithms for short- range molecular dynamics. *J. Comput. Phys.* **117**, 44 (1995).

39. G. Nikoulis, P. Grammatikopoulos, S. Steinhauer, J. Kioseoglou, NanoMaterialsCAD: Flexible software for the design of nanostructures. *Adv. Theory Simul.* **4**, 2000232 (2021).

40. P. K. Schelling, S. R. Phillpot, P. Keblinski, Comparison of atomic-level simulation methods for computing thermal conductivity. *Phys. Rev. B* **65**, 144306 (2002).

41. K. Fuchs, The conductivity of thin metallic films according to the electron theory of metals. *Math. Proc. Camb. Philos.* **34**, 100–108 (1938).

42. E. H. Sondheimer, The mean free path of electrons in metals. *Adv. Phys.* **1**, 1–42 (1952).

43. A. J. Minnich, Determining phonon mean free paths from observations of quasiballistic thermal transport. *Phys. Rev. Lett.* **109**, 205901 (2012).

44. E. Chavez-Angel, R. A. Zarate, S. Fuentes, E. J. Guo, M. Kläui, G. Jakob, Reconstruction of an effective magnon mean free path distribution from spin Seebeck measurements in thin films. *New J. Phys.* **19**, 013011 (2017).

45. M.-Á. Sanchez-Martinez, F. Alzina, J. Oyarzo, C. M. Sotomayor Torres, E. Chavez-Angel, Impact of the regularization parameter in the mean free path reconstruction method: Nanoscale heat transport and beyond. *Nanomaterials* **9**, 414 (2019).

46. S. Alaie, D. F. Goettler, M. Su, Z. C. Leseman, C. M. Reinke, I. El-Kady, Thermal transport in phononic crystals and the observation of coherent phonon scattering at room temperature. *Nat. Commun.* **6**, 7228 (2015).

47. M. Kasprzak, M. Sledzinska, K. Zaleski, I. Iatsunskyi, F. Alzina, S. Volz, C. M. Sotomayor Torres, B. Graczykowski, High-temperature silicon thermal diode and switch. *Nano Energy* **78**, 105261 (2020).

48. M. Nomura, R. Anufriev, Z. Zhang, J. Maire, Y. Guo, R. Yanagisawa, S. Volz, Review of thermal transport in phononic crystals. *Mater. Today Phys.* **22**, 100613 (2022).

49. R. Anufriev, Y. Wu, J. Ordonez-Miranda, M. Nomura, Nanoscale limit of the thermal conductivity in crystalline silicon carbide membranes, nanowires, and phononic crystals. *NPG Asia Mater* **14**, 35 (2022).

50. R. Anufriev, J. Maire, M. Nomura, Reduction of thermal conductivity by surface scattering of phonons in periodic silicon nanostructures. *Phys. Rev. B* **93**, 045411 (2016).

51. M. Sledzinska, B. Graczykowski, F. Alzina, U. Melia, K. Termentzidis, D. Lacroix, C. M. Sotomayor Torres, Thermal conductivity in disordered porous nanomembranes. *Nanotechnology* **30**, 265401 (2019).

52. K. Takahashi, M. Fujikane, Y. Liao, M. Kashiwagi, T. Kawasaki, N. Tambo, S. Ju, Y. Naito, J. Shiomi, Elastic inhomogeneity and anomalous thermal transport in ultrafine Si phononic crystals. *Nano Energy* **71**, 104581 (2020).

53. J.-K. Yu, S. Mitrovic, D. Tham, J. Varghese, J. R. Heath, Reduction of thermal conductivity in phononic nanomesh structures. *Nat. Nanotech.* **5**, 718–721 (2010).

54. M. Verdier, D. Lacroix, S. Didenko, J.-F. Robillard, E. Lampin, T.-M. Bah, K. Termentzidis, Influence of amorphous layers on the thermal conductivity of phononic crystals. *Phys. Rev. B* **97**, 115435 (2018).

55. M. Ghorbani-Asl, S. Kretschmer, D. E. Spearot, A. V. Krasheninnikov, Two-dimensional MoS_2 under ion irradiation: From controlled defect production to electronic structure engineering. *2D Mater.* **4**, 025078 (2017).

56. S. Ghaderzadeh, V. Ladygin, M. Ghorbani-Asl, G. Hlawacek, M. Schleberger, A. V. Krasheninnikov, Freestanding and supported MoS_2 monolayers under cluster irradiation: Insights from molecular dynamics simulations. *ACS Appl. Mater. Interfaces* **12**, 37454–37463 (2020).

57. G. J. Snyder, M. Soto, R. Alley, D. Koester, B. Conner, Hot spot cooling using embedded thermoelectric coolers, in *Twenty-Second Annual IEEE Semiconductor Thermal Measurement And Management Symposium (IEEE)* (IEEE, 2006), pp. 135–143.

58. K. Xu, A. J. Gabourie, A. Hashemi, Z. Fan, N. Wei, A.B. Farimani, H.P.Komsa, A. V. Krasheninnikov, E. Pop, T. Ala-Nissila, Thermal transport in MoS_2 from molecular dynamics using different empirical potentials. *Phys. Rev. B* **99**, 054303 (2019).

59. D.J. Evans, Homogeneous NEMD algorithm for thermal conductivity—Application of non-canonical linear response theory. *Phys. Lett. A* **91**, 457–460 (1982).

60. D.J. Evans, G.P. Morriss, *Statistical Mechanics of Nonequilibrium Liquids* (Academic Press, ed. 1, 1990).

61. Z. Fan, H. Dong, A. Harju, T. Ala-Nissila, Homogeneous nonequilibrium molecular dynamics method for heat transport and spectral decomposition with many-body potentials. *Phys. Rev. B* **99**, 064308 (2019).
